# Supplementary material for: Communicating benign biopsy results by telephone in the NHS Breast Screening Programme: a protocol for a cluster randomised crossover trial
Source: BMJ Open. 2019 Aug 2;9(8):e028679. doi: 10.1136/bmjopen-2018-028679 (PMC6687008; doi:10.1136/bmjopen-2018-028679)
Supplement: Supplementary data [file bmjopen-2018-028679supp001.pdf]

Survey (time 1) – V.6 05.04.2018

# Communicating results in breast screening: Research survey – Time 1

Public Health England

WARWICK
THE UNIVERSITY OF WARWICK

NHS
Cancer Screening Programmes

**PARTICIPANT NAME: ________________________________**

(Please ensure this is completed)

**For researcher use only:**

Participant ID number: ________________________________

B1 or B2 result:

- ☐ Yes
- ☐ No



Williamson SZ, *et al. BMJ Open* 2019; 9:e028679. doi: 10.1136/bmjopen-2018-028679

Survey (time 1) – V.6 05.04.2018

**Participant information**

**1) What is your age?**

____________ years

**2) Have you attended screening before?**

☐ Yes
☐ No

**2a) If yes, did you have a biopsy (a needle inserted into the breast to remove tissue)?**

☐ Yes
☐ No
☐ Can't remember

**3) Please tick the highest level of education you have achieved:**

☐ No schooling completed
☐ High school or equivalent
☐ Some college
☐ Bachelor's degree
☐ Master's degree
☐ Doctoral degree
☐ Professional degree
☐ Other

**4) Please tick your marital status:**

☐ Single
☐ Domestic partnership
☐ Married
☐ Divorced
☐ Widowed
☐ Separated

**5) What is your ethnic group?**

White:

☐ English/Welsh/Scottish/Northern Irish/British
☐ Irish
☐ Gypsy or Irish Traveller
☐ Any other White background

Asian/Asian British:

☐ Indian
☐ Pakistani
☐ Bangladeshi
☐ Chinese
☐ Any other Asian background

Mixed/multiple ethnic groups:

☐ White and Black Caribbean
☐ White and Black African
☐ White and Asian
☐ Any other Mixed/Multiple ethnic background

Black/African/Caribbean/Black British:

☐ African
☐ Caribbean
☐ Any other Black/African/Caribbean background

Other ethnic group:

☐ Arab
☐ Any other ethnic group

Survey (time 1) – V.6 05.04.2018

**Preferences for communication**

When a screening result is not cancer, some centres deliver these results in-person and some deliver these results over the telephone. We want to know how you would prefer to receive screening results, if you were given the choice.

Please note, if you tick a preference, you may not necessarily get your result delivered this way.

**6) How would you prefer to receive your results from screening?**

☐ In-person at the screening centre
☐ By telephone

**7) Why would you prefer to receive your results this way?**

Please write a brief explanation:

____________________________________________________________

____________________________________________________________

____________________________________________________________



Williamson SZ, *et al. BMJ Open* 2019; 9:e028679. doi: 10.1136/bmjopen-2018-028679

Survey (time 1) – V.6 05.04.2018

## How do you feel?

**8) Over the last week how often have you experiences the following things because of thoughts and feelings about breast cancer:**

| | Not at all | Rarely | Some of the time | Quite a lot of the time |
|---|---|---|---|---|
| Had trouble sleeping | 0 | 1 | 2 | 3 |
| Experienced a change in appetite | 0 | 1 | 2 | 3 |
| Been unhappy or depressed | 0 | 1 | 2 | 3 |
| Been scared and panicky | 0 | 1 | 2 | 3 |
| Felt nervous or strung up | 0 | 1 | 2 | 3 |
| Felt under strain | 0 | 1 | 2 | 3 |
| Found you have been keeping things from those who are close to you | 0 | 1 | 2 | 3 |
| Found yourself taking things out on other people | 0 | 1 | 2 | 3 |
| Found yourself noticeably withdrawing from those who are close to you | 0 | 1 | 2 | 3 |
| Had difficultly doing things around the house that you normally do | 0 | 1 | 2 | 3 |
| Had difficulty meeting work or other commitments | 0 | 1 | 2 | 3 |
| Felt worried about your future | 0 | 1 | 2 | 3 |



Williamson SZ, *et al. BMJ Open* 2019; 9:e028679. doi: 10.1136/bmjopen-2018-028679

Survey (time 1) – V.6 05.04.2018

**9) Please provide a contact name and address for follow-up surveys to be sent:**

(Please note that this address will remain anonymous and all contact information will be destroyed once the study is complete. This address will only be used for the purposes of this research to post follow-up surveys.)

________________________________________

________________________________________

________________________________________

________________________________________

________________________________________

**10) Please provide a contact telephone number:**

(Please note that this contact number will remain anonymous and all contact information will be destroyed once the study is complete. This telephone number will only be used for the purposes of this research to remind you of the first follow-up survey.)

________________________________________

**11) Would you like to hear about the results from this research?**

(If you choose yes, you will be sent a copy of the results from this research via the address provided)

☐ Yes
☐ No
