# Supplementary material for: Communicating benign biopsy results by telephone in the NHS Breast Screening Programme: a protocol for a cluster randomised crossover trial
Source: BMJ Open. 2019 Aug 2;9(8):e028679. doi: 10.1136/bmjopen-2018-028679 (PMC6687008; doi:10.1136/bmjopen-2018-028679)
Supplement: Author's manuscript [file bmjopen-2018-028679.draft_revisions.pdf]

BMJ Open is committed to open peer review. As part of this commitment we make the peer review history of every article we publish publicly available.

When an article is published we post the peer reviewers' comments and the authors' responses online. We also post the versions of the paper that were used during peer review. These are the versions that the peer review comments apply to.

The versions of the paper that follow are the versions that were submitted during the peer review process. They are not the versions of record or the final published versions. They should not be cited or distributed as the published version of this manuscript.

BMJ Open is an open access journal and the full, final, typeset and author-corrected version of record of the manuscript is available on our site with no access controls, subscription charges or pay-per-view fees (http://bmjopen.bmj.com).

If you have any questions on BMJ Open's open peer review process please email info.bmjopen@bmj.com

BMJ Open

Communicating benign biopsy results by telephone in the NHS Breast Screening Programme: a protocol for a randomised controlled trial

Journal:	*BMJ Open*
Manuscript ID	bmjopen-2018-028679
Article Type:	Protocol
Date Submitted by the Author:	19-Dec-2018

Complete List of Authors:	Williamson, Sian; University of Warwick, Health Sciences<br>Johnson, Rebecca; University of Warwick,<br>Sandhu, Harbinder; University of Warwick Warwick Medical School, Clinical Trials Unit<br>Parsons, Nicholas; University of Warwick, Warwick Medical School<br>Jenkins, Jacquie; Public Health England, National Programme Manager – NHS Breast Screening Programme<br>Casey, Margaret; Royal Wolverhampton NHS Trust, Clinical Nurse Specialist Breast Care<br>Kearins, Olive; Public Health England, National Lead Breast Screening QA<br>Taylor-Phillips, Sian; University of Warwick, Warwick Medical School

Keywords:	PREVENTIVE MEDICINE, Health policy < HEALTH SERVICES ADMINISTRATION & MANAGEMENT, Protocols & guidelines < HEALTH SERVICES ADMINISTRATION & MANAGEMENT, Quality in health care < HEALTH SERVICES ADMINISTRATION & MANAGEMENT

SCHOLARONE™
Manuscripts

Protocol paper V.6 19.12.2018

For peer review only

Communicating benign biopsy results by telephone in the NHS Breast Screening Programme: a protocol for a randomised controlled trial

Sian Williamson, s.williamson@warwick.ac.uk (PhD Student, University of Warwick),

Rebecca Johnson, rebecca.johnson@warwick.ac.uk (Senior Research Fellow, University of Warwick),

Harbinder Sandhu, harbinder.k.sandhu@warwick.ac.uk (Associate Professor, University of Warwick),

Nick Parsons, nick.parsons@warwick.ac.uk (Statistician, Principal Research Fellow, University of Warwick)

Jacquie Jenkins, jacquie.jenkins@phe.gov.uk (National Programme Manager – NHS Breast Screening Programme),

Margaret Casey, margaret.casey@nhs.net (Clinical Nurse Specialist Breast Care),

Olive Kearins, Olive.Kearins@phe.gov.uk (National Lead Breast Screening QA, Public Health England)

& Sian Taylor-Phillips, s.taylor-phillips@warwick.ac.uk (Associate Professor, University of Warwick)

Corresponding author: Sian Taylor-Phillips

Word count (excluding abstract): 3,361 words

Keywords: Mass screening, communication, cancer screening, RCT

Protocol paper V.6 19.12.2018

Abstract

Introduction – One of the main harms from breast cancer screening is the anxiety caused by false positive results. Various factors may be associated with false-positive anxiety. One modifiable factor may be the method of communication used to deliver results. The aim of this study is to measure the effect on anxiety of receiving benign biopsy results in-person or by telephone.

Methods and analysis – This is a multi-centre randomised controlled trial in the English NHS Breast Screening Programme (NHSBSP) involving repeated survey measures at four time points. Participants will be women of screening age attending the NHSBSP for further tests following a suspicious mammography result. Centres will be randomised to deliver results in-person or by telephone. Women will be blinded to the method of communication.

The analysis will compare women who have received telephone results and women who have received in-person results. The primary outcome measure will be anxiety after receiving results, whilst controlling for baseline anxiety. Secondary outcome measures will include anxiety at 3 and 6 months post-results, understanding of results and patient preferences for how results are communicated. Qualitative interviews will also be conducted to further explore women's reasons for communication preferences. Qualitative and quantitative data will be integrated after initial separate analysis using the Pillar Integration Process.

Ethics and dissemination – This study has been approved by the Public Health England Breast Screening Programme Research Advisory Committee, (BSPRAC_0013, ODR1718_040) and the HRA NHS West Midlands - Coventry & Warwickshire Research Ethics Committee (17/WM/0313). The findings from this study will be disseminated to key stakeholders within the NHSBSP and via academic publications.

Registration details – ISRCTN36997684 https://doi.org/10.1186/ISRCTN36997684

For peer review only

Protocol paper V.6 19.12.2018

Article summary

Strengths and limitations of this study

- This is the first randomised controlled trial to measure the impact of communication method on patient outcomes when delivering results in breast screening
- The mixed-methods design adds depth to our understanding of communication preferences and the mechanisms by which anxiety may be affected
- The study is in English breast screening centres and generalisability to other contexts should be carefully considered

For peer review only

Protocol paper V.6 19.12.2018

Introduction

The UK National Health Service Breast Screening Programme (NHSBSP) is a population-based screening programme that aims to detect breast cancer earlier. Asymptomatic women aged 50-70 are invited to attend every three years for a mammogram. If a suspected abnormality is found on the mammogram then the woman is invited back to the clinic for follow-up tests. Follow-up tests can include clinical examination, a further mammogram or ultrasound. If these indicate suspicion of cancer then the woman receives a core needle biopsy, involving the removal of sections of tissue from the suspicious breast region. This tissue can then be pathologically analysed. Although the procedure is designed to be minimally invasive, leaving only minor bruising, some women find the experience painful and distressing. Results of diagnostic tests are discussed on a case-by-case basis at multidisciplinary team meetings (MDT) using a triple-assessment of clinical examination, imaging and biopsy report.

A false-positive result is when a woman has been identified at the screening phase as potentially having cancer, but follow up tests have revealed no abnormalities. Receiving a false-positive result from screening is very common, with the majority of women who are recalled ultimately being given a false-positive result (1). In England, around half of women receive the all clear results at the time of the follow-up tests but the other half require a biopsy and subsequent histological analysis. These women receive their results at a later date, either via telephone or in-person.

Screening programmes should provide benefit that outweighs both physical and psychological harm (2, 3). One of the main harms from breast screening is the anxiety caused by false positive results (4). Women who are invited to be screened have no symptoms of breast cancer at the time of their initial mammogram. Telling a woman that something suspicious has been found in the mammogram and that further tests are needed can make her feel very anxious and believe that she might have cancer (5). For some women, once results confirm the absence of cancer, anxiety declines. However, anxiety can remain elevated for much longer, lasting up to three years after receiving the benign result and leading into the next screening invitation (6). This is an issue, as the NHSBSP has a duty to minimize the harm caused by screening.

For peer review only

There are various factors that may be associated with heightened anxiety during screening such as family history, lower education, younger age and individual differences in personality (7-12). These factors are unmodifiable and tend to be focused at the level of the individual, which makes the minimisation of screening anxiety a challenge. However, it is possible that there are modifiable changes that can be made at the organisational level of screening that may minimise the impact of anxiety. One of these changes is the method of communication used to deliver results.

NHSBSP guidelines for communicating results state that telephone results 'should not be routinely offered' (13). However, most breast screening centres in the UK deliver benign results to the woman over the telephone. Some breast care nurses remain concerned about how the communication method used to deliver results may contribute to the anxiety experienced by women attending screening (14). Telephone results may offer advantages, eliminating the stress and costs associated with transport, parking and anxiously waiting in a clinic for results. However, telephone results eliminates the in-person encounter, meaning all communication is verbal only. Research from other areas has shown that non-verbal communication plays a key role in enhancing understanding and minimising anxiety.

The impact of the communication methods used to deliver benign results in the NHSBSP has not yet been explored.

For peer review only

Protocol paper V.6 19.12.2018

Aim of the study

The aim of this study is to compare anxiety in women receiving benign biopsy results from the NHSBSP via telephone results or in-person.

Methods and analysis

Study design

Patient and public involvement from the charity Independent Cancer Patient Voices was used to guide the design of the study to ensure the acceptability of the method and appropriateness of the participant materials.

The study design chosen was a multi-centre randomised controlled trial. The randomisation allows for the direct comparison of study outcomes between women who received telephone results and women who received in-person results, whilst controlling for confounding factors such as education, age and individual differences in baseline anxiety.

Participants and settings

This study will be a prospective, longitudinal randomised controlled trial conducted in a breast screening centre setting. The study will take place in the UK where women are invited to attend breast screening every 3 years for digital mammography. The study will take place across four time points (see Table 1) with a survey completed by participants at each stage. The study participants will be recruited from 5 English Breast Screening centres across different regions.

Participants will be women between the ages of 47-73 attending the NHSBSP for further tests following a suspicious mammogram. This includes women offered routine screening between ages 50-70 and those receiving extra rounds of screening between the ages of 47-50 or 71-73 as part of the UK age extension trial (15). Women will be recruited at the assessment clinic pre-biopsy. However, only women who have receive a benign (B2) or normal (B1) biopsy result will be included in the longitudinal data collection. To participate, women must have English as a first or second language and have the capacity to consent.

For peer review only

Table 1: Time points for the study

Time point	Sample	Survey content	Method
Time 1 – at assessment clinic	Women attending screening follow-up clinic	Demographic information<br>Communication preferences<br>Baseline anxiety score (PCQ)<br>Contact details	In-person
Time 2 – after receiving results	Women from time 1 who had a normal or benign biopsy	Communication preferences<br>Repeat anxiety score (PCQ)<br>Measure of understanding	By post
Time 3 – Three month follow-up	Women from time 1 who had a normal or benign biopsy	Repeat anxiety score (PCQ)	By post
Time 4 – Six month follow-up	Women from time 1 who had a normal or benign biopsy	Repeat anxiety score (PCQ)	By post

Measuring anxiety

The Psychological Consequences Questionnaire (PCQ) was selected as the most appropriate measure of anxiety in the breast screening setting (16). The PCQ is a disease specific measure, focusing on breast cancer specific anxiety. This avoids the contrast-validity that is associated with using generic anxiety measures in the breast screening context (17). The PCQ is widely used in the breast screening setting (6, 8, 18-20).

Participants will rate their anxiety on the PCQ by judging each statement on a scale of 0 (not at all) to 3 (quite a lot of the time). Women will be asked "*Over the last week* how often have you experiences the following things because of *thoughts and feelings about breast cancer*:"

For peer review only

Sample size considerations

The sample size was calculated based on the primary outcome, which is anxiety score at time point 2. The aim is to be able to detect a clinically significant difference of 3 points on the PCQ (the difference between the score on one statement being 0, not at all, and 3, quite a lot of the time).

[Insert Figure 1 here]

Figure 1: Flow of required sample size for the study

Assuming a 5% significance and 80% power, 194 participants are required at time 2, with 97 participants per arm. Allowing for attrition rate between time point 1 and time point 2 due to participant withdrawal (15%) and participant eligibility (50%), a total of 457 participants will be recruited at time point 1.

A design effect was applied, inflating the sample size in order to be conservative, but not in excess. The ICC was estimated to be 0.01. The number of observations within each cluster were assumed to be equal. In this case, the cluster was the number of centres, 4. With the sample size of 194 women, divided by the number of centres (4) and then divided by the number of interventions (2), this led to the number of observations within a cluster being 48.2. This gave a design effect of 1.49. Taking this into account, the sample size needed to achieve statistical significance (194 women) was multiplied by the design effect, giving a total sample of 290 women at time point 2 when rounded up.

31,926 women are recalled for a biopsy each year. If half of the 31,926 biopsies come back as benign, this leaves a potential sample of 15,963. This means that, on average across 80 breast screening centres, each centre will have around 200 benign biopsy results each year. Therefore, assuming 50% participation rates of eligible women, recruitment will require four centres for one year.

For peer review only

Randomisation and blinding

Each centre will be randomised to one of two arms (see Table 2). Each centre will commence with the communication method randomised in month 1 and continue to alternative between the two communication methods for the duration of the study.

Table 2: Allocation of communication method by arm of the trial

Month	Arm 1	Arm 2
1	Telephone	In-person
2	In-person	Telephone
3	Telephone	In-person
4	In-person	Telephone
5	Telephone	In-person
6	In-person	Telephone

Women will receive their results in accordance with the month in which they attend the recall clinic but will be blinded to which communication method will be used. This design was chosen to ensure balance between trial arms at each site whilst addressing practical constraints. Balance between trial arms at each site is important to account for centre level confounders such as staff communication skills and centre level processes.

Individual randomisation was not possible as it interfered with screening centres workflow and fail-safe mechanisms to ensure that every woman was contacted with the correct information within the correct time frame. We randomly assigned whether the first month at each centre telephone or in-person.

Participants will be blinded to the randomisation month. Breast screening staff will not be blinded, as they will be delivering the results and scheduling the appointments.

For peer review only

Protocol paper V.6 19.12.2018

Allocation of communication method

There are two types of centres who may be involved in the research: centres who currently deliver benign results in-person and centres who currently deliver benign results by telephone.

For centres currently delivering results in-person, the following process for scheduling a results appointment will be observed: During in-person study months, all consenting women at time 1 will be given an appointment to re-attend to receive their results in-person. During telephone study months, all consenting women at time 1 will be given an appointment to re-attend to receive their results in person. However, they will consent to potentially receiving results over the telephone, which will happen prior to their scheduled appointment. Only benign women will be telephoned, with all women receiving other results attending their scheduled appointment

For centres currently delivering results by telephone, the following process for scheduling a results appointment will be observed: All consenting women will be informed that, when their results are ready, they will be contacted by telephone to arrange an appointment to come back for their results. During the 'telephone' months of the study, women will be telephoned as expected. However, instead of arranging an appointment during this telephone call, results will be delivered. This means that, for women who go on to have a cancer result, they can be telephoned to arrange an appointment to attend in-person, as expected. This is in-line with standard practice at these centres. During the in-person months, all consenting women will be informed that, when their results are ready, they will be telephoned to arrange an appointment to come back for their results. These women will be telephoned to arrange an appointment to come back in-person, and at that appointment they will receive their benign/normal result.

All women not enrolled in the study will receive their screening result based on the current standard practice at the attended centre.

For peer review only

Protocol paper V.6 19.12.2018

Data collection

Time point 1

Participant recruitment at time one will occur concurrently at each breast screening centre.

Women will be approached during their assessment visit by breast care nurses with Good Clinical Practice (GCP) training. These women will have been recalled from a previous mammogram and may have a biopsy as part of the assessment clinic. The study will be explained to potential participants and nurses will go through the informed consent process. Consenting women will fill out the Time point 1 survey during their follow-up visit, with study responses collected and stored securely before the participant leaves the clinic.

Multi-disciplinary team meeting

At the local level multi-disciplinary team meeting for breast screening staff, women recruited into the study will be included in further time points if they receive a benign (B2) or normal (B1) result. A breast care review will compile the contact details of eligible women into a spreadsheet to be sent securely to the research team.

Time point 2

The research team will distribute Time point 2 surveys to eligible women with a pre-paid return envelope. If no response is received within a week, the research team will contact women by telephone as a reminder. A maximum of two telephone contact attempts will be made. This is to ensure anxiety and understanding are measured at the crucial post-results stage of the screening process.

As part of Time point 2 survey, women will be asked if they would like to participate in further research involving an interview about their experience of receiving a screening result.

For peer review only

Protocol paper V.6 19.12.2018

Time point 3

The research team will distribute Time point 3 surveys, three months after the biopsy result was received.

Time point 4

The research team will distribute Time point 4 surveys, six months after the biopsy result was received.

Qualitative interviews

The qualitative interviews will explore why women prefer certain methods of communication. Women will be recruited from the time point 2 survey. Women who express an interest in participating will be sent further information about the interviews. If they wish to participate, women will return the consent form in the pre-paid envelope. Women will then be contacted by the research team to be interviewed.

The semi-structured interviews will each take 10-20 minutes. Questions will encompass the woman's experience of receiving a result from screening. This will involve asking how the woman felt, whether she understood her result and an exploration of her views on different methods for communicating results. Interviews will be audio-recorded and transcribed verbatim. Data collection will cease once no further themes emerge and data saturation is reached.

Mixed methods integration

Using a mixed methods approach, the quantitative preference survey data will be combined with the findings from the qualitative interviews. The quantitative data asks about women's communication preferences, whilst the qualitative interviews expand on this by exploring how women justify certain preferences for communication. In mixing the data, the qualitative data will be used to *expand* the understanding of the findings from the quantitative surveys (21). Expansion provides richness and detail, expanding why and how women form communication preferences and moving understanding of preferences *beyond* what quantitative data or qualitative data in isolation can elucidate. The value added by integrating the knowledge of both *what*

For peer review only

women prefer and *why* is in the completeness of our understanding of preferences, making the evidence that will inform the NHSBSP policy decisions comprehensive and patient-centred.

For peer review only

Outcomes and study measures (primary outcome, secondary outcomes)

Primary outcome

The primary outcome is the PCQ anxiety score at time point 2.

A comparison in anxiety score will be made between women who receive results in-person and women who receive results over the telephone.

Secondary outcomes

Secondary outcomes are:

- PCQ anxiety score at 3 month follow-up (time 3)
- PCQ anxiety score at 6 month follow-up (time 4)
- Subjective understanding of results (time 2)
    - Measured using a survey question designed in collaboration with NHSBSP stakeholders
- Objective understanding of results (time 2)
    - Measured using a survey question designed in collaboration with NHSBSP stakeholders
- Quantitative preferences for results communication before results (time 1)
- Quantitative preferences for results communication after results (time 2)
- Qualitative preferences for results communication before results (time 1)
- Qualitative preferences for results communication after results (time 2)

Analysis

Quantitative data - Statistical analysis

Primary outcome

The primary analysis will use a mixed effects model to estimate the effects of communication method on anxiety (time 2), after adjusting for baseline (time 1). Fixed explanatory effects will include age, ethnicity, previous screening attendance, previous biopsy education and marital status.

For peer review only

Protocol paper V.6 19.12.2018

Statistical significance will be assessed at the 5% level.

Secondary outcomes

Longitudinal anxiety scores at 3 and 6 months will be analysed in the same way as the primary anxiety outcome.

Differences in understanding score between communication methods groups will be assessed using a logistic regression model, adjusting for age, ethnicity, previous attendance, previous biopsy, education, marital status.

Preference data from the quantitative surveys will be presented in the form of percentages. Preference data from the qualitative interviews will be analysed using inductive thematic analysis, managed using NVivo10. A mixed-methods approach will then be taken to integrate the quantitative data on patient preferences and the qualitative data on reasons for these preferences (22).

<u>Qualitative data analysis</u>

Qualitative preference data from the time point 1 and time point 2 surveys and data from the telephone interviews will be analysed using inductive thematic analysis (23), managed using NVivo10.

<u>Mixed-methods integration</u>

To integrate the quantitative and qualitative data, the Pillar Integration Process will be used (22). This analytical integration technique uses four systematic stages (listing, matching, checking and pillar-building) to identify and examine connections and discrepancies in qualitative and quantitative findings. It allows for the visual display of the data and findings; this enhances overall transparency of the integration approach and the results of such an integration.

<u>Ethics</u>

Attention was given to the various ethical challenges of the trial. The main ethical issue will be the use of sensitive patient information (addresses, telephone

For peer review only

numbers). Participants will be told explicitly how their contact details will be used and stored throughout the data collection process. Participants will give informed consent for their contact details to be used for the purposes of the study.

All electronic data will be transferred securely in a password protected excel document from secure email accounts. All raw survey data will be collected directly from the centres by the lead researcher and be transferred in a secure lock-box.

Ethical approval for this study was granted by the following organisations:

- Public Health England Breast Screening Programme Research Advisory Committee, BSPRAC_0013 (ODR1718_040)
- HRA NHS West Midlands - Coventry & Warwickshire Research Ethics Committee (17/WM/0313)

Dissemination

Results from the trial will disseminated directly to key stakeholders within the NHSBSP. This will encourage discussion regarding how benign results are communicated in breast screening, and how this might best be implemented in order to minimise the anxiety women experience.

The results will also be disseminated via academic publications.

For peer review only

Protocol paper V.6 19.12.2018

Current study status

The trial began recruitment in February 2018. Data collection is due to conclude in March 2019. The trial statisticians (SW and NP) have received recruitment data but no results will be transferred to the statisticians until recruitment is closed in March 2019.

Author contributions

SZW is the lead researcher. SZW and STP drafted the manuscript. SZW, RJ, HS and STP participated in the design of the study. JJ, MC and OK are key stakeholders in the NHSBSP who assisted with the study design. NP aided with the statistical analysis. All authors have reviewed and approved the manuscript.

Funding statement

This research is part of a PhD award and is funded by the Economic and Social Research Council (ESRC) Doctoral Training Centre at the University of Warwick. The funding has been awarded for this studentship to Sian Williamson for her PhD project for 4 years of full-time study. The award consists of payment of academic fees and a maintenance award.

A further contract between the University of Warwick, Public Health England (PHE) and the PhD Student (Sian Williamson) has secured £4,000 in research expenses.

Competing interests

None declared

For peer review only

Protocol paper V.6 19.12.2018

References

1. DeFrank JT, Brewer N. A model of the influence of false-positive mammography screening results on subsequent screening. Health psychology review. 2010;4(2):112-27.
2. Raffle AE, Gray JM. Screening: evidence and practice: Oxford University Press; 2007.
3. Marmot MG, Altman D, Cameron D, Dewar J, Thompson S, Wilcox MJBjoc. The benefits and harms of breast cancer screening: an independent review. 2013;108(11):2205.
4. Bond M, Pavey T, Welch K, Cooper C, Garside R, Dean S, et al. Systematic review of the psychological consequences of false-positive screening mammograms. 2013.
5. Solbjør M, Forsmo S, Skolbekken J-A, Sætnan AR. Experiences of recall after mammography screening—a qualitative study. Health care for women international. 2011;32(11):1009-27.
6. Brett J, Austoker J. Women who are recalled for further investigation for breast screening: psychological consequences 3 years after recall and factors affecting re-attendance. Journal of Public Health. 2001;23(4):292-300.
7. Matteau TM. Psychology and screening: narrowing the gap between efficacy and effectiveness. British Journal of Clinical Psychology. 1994;33(1):1-10.
8. Brett J, Bankhead C, Henderson B, Watson E, Austoker J. The psychological impact of mammographic screening. A systematic review. Psycho-Oncology. 2005;14(11):917-38.
9. Ando N, Iwamitsu Y, Kuranami M, Okazaki S, Nakatani Y, Yamamoto K, et al. Predictors of psychological distress after diagnosis in breast cancer patients and patients with benign breast problems. Psychosomatics. 2011;52(1):56-64.
10. Keyzer-Dekker CM, de Vries J, Mertens MC, Roukema JA, van der Steeg AF. Cancer or no cancer: the influence of trait anxiety and diagnosis on quality of life with breast cancer and benign disease: a prospective, longitudinal study. World journal of surgery. 2013;37(9):2140-7.
11. Montgomery M, McCrone SH. Psychological distress associated with the diagnostic phase for suspected breast cancer: systematic review. Journal of advanced nursing. 2010;66(11):2372-90.
12. Sweeny K, Andrews SE. Mapping individual differences in the experience of a waiting period. Journal of personality and social psychology. 2014;106(6):1015.
13. England. PH. NHS public health functions agreement 2017- 18 Service specification no.24 Breast Screening Programme. In: England. PH, editor. 2017.
14. Chapman K. Interim quality assurance guidelines for clinical nurse specialists in breast cancer screening. 2012.
15. 2000. CgIBMNLoMUF. Identifier NCT01081288; Extending the National Health Service (NHS) Breast Screening Age Range (AgeX). 5 March 2010 (16 November 2018).
16. Cockburn J, De Luise T, Hurley S, Clover K. Development and validation of the PCQ: a questionnaire to measure the psychological consequences of screening mammography. Social science & medicine. 1992;34(10):1129-34.
17. Brodersen J, Thorsen H, Cockburn J. The adequacy of measurement of short and long-term consequences of false-positive screening mammography. Journal of medical screening. 2004;11(1):39-44.

For peer review only

Protocol paper V.6 19.12.2018

18. Sweeny K, Christianson D, McNeill J. The Psychological Experience of Awaiting Breast Diagnosis. 2018.
19. Cockburn J, Staples M, Hurley SF, De Luise T. Psychological consequences of screening mammography. Journal of Medical Screening. 1994;1(1):7-12.
20. Brewer NT, Salz T, Lillie SE. Systematic Review: The Long-Term Effects of False-Positive MammogramsThe Long-Term Effects of False-Positive Mammograms. Annals of internal medicine. 2007;146(7):502-10.
21. Creswell JW, Klassen AC, Plano Clark VL, Smith KC. Best practices for mixed methods research in the health sciences. Bethesda (Maryland): National Institutes of Health. 2011:2094-103.
22. Johnson RE, Grove AL, Clarke AJJoMMR. Pillar Integration Process: A Joint Display Technique to Integrate Data in Mixed Methods Research. 2017:1558689817743108.
23. Braun V, Clarke VJQrip. Using thematic analysis in psychology. 2006;3(2):77-101.

For peer review only

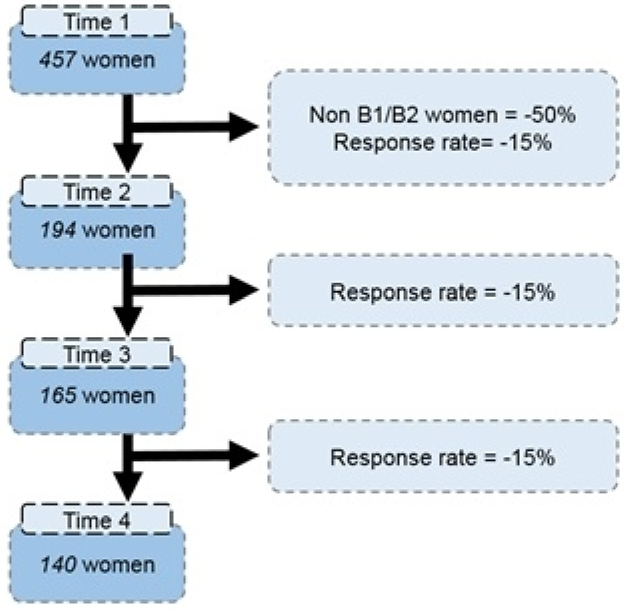

Figure 1 - Flow of required sample size for the study

85x84mm (96 x 96 DPI)

SPIRIT

STANDARD PROTOCOL ITEMS: RECOMMENDATIONS FOR INTERVENTIONAL TRIALS

SPIRIT 2013 Checklist: Recommended items to address in a clinical trial protocol and related documents*

Section/item	Item No	Description
**Administrative information**		
Title	1	Descriptive title identifying the study design, population, interventions, and, if applicable, trial acronym
Trial registration	2a	Trial identifier and registry name. If not yet registered, name of intended registry
	2b	All items from the World Health Organization Trial Registration Data Set
Protocol version	3	Date and version identifier
Funding	4	Sources and types of financial, material, and other support
Roles and responsibilities	5a	Names, affiliations, and roles of protocol contributors
	5b	Name and contact information for the trial sponsor
	5c	Role of study sponsor and funders, if any, in study design; collection, management, analysis, and interpretation of data; writing of the report; and the decision to submit the report for publication, including whether they will have ultimate authority over any of these activities
	5d	Composition, roles, and responsibilities of the coordinating centre, steering committee, endpoint adjudication committee, data management team, and other individuals or groups overseeing the trial, if applicable (see Item 21a for data monitoring committee)
**Introduction**		
Background and rationale	6a	Description of research question and justification for undertaking the trial, including summary of relevant studies (published and unpublished) examining benefits and harms for each intervention
	6b	Explanation for choice of comparators
Objectives	7	Specific objectives or hypotheses
Trial design	8	Description of trial design including type of trial (eg, parallel group, crossover, factorial, single group), allocation ratio, and framework (eg, superiority, equivalence, noninferiority, exploratory)

For peer review only

Methods: Participants, interventions, and outcomes

Study setting	9	Description of study settings (eg, community clinic, academic hospital) and list of countries where data will be collected. Reference to where list of study sites can be obtained
Eligibility criteria	10	Inclusion and exclusion criteria for participants. If applicable, eligibility criteria for study centres and individuals who will perform the interventions (eg, surgeons, psychotherapists)
Interventions	11a	Interventions for each group with sufficient detail to allow replication, including how and when they will be administered
	11b	Criteria for discontinuing or modifying allocated interventions for a given trial participant (eg, drug dose change in response to harms, participant request, or improving/worsening disease)
	11c	Strategies to improve adherence to intervention protocols, and any procedures for monitoring adherence (eg, drug tablet return, laboratory tests)
	11d	Relevant concomitant care and interventions that are permitted or prohibited during the trial
Outcomes	12	Primary, secondary, and other outcomes, including the specific measurement variable (eg, systolic blood pressure), analysis metric (eg, change from baseline, final value, time to event), method of aggregation (eg, median, proportion), and time point for each outcome. Explanation of the clinical relevance of chosen efficacy and harm outcomes is strongly recommended
Participant timeline	13	Time schedule of enrolment, interventions (including any run-ins and washouts), assessments, and visits for participants. A schematic diagram is highly recommended (see Figure)
Sample size	14	Estimated number of participants needed to achieve study objectives and how it was determined, including clinical and statistical assumptions supporting any sample size calculations
Recruitment	15	Strategies for achieving adequate participant enrolment to reach target sample size

Methods: Assignment of interventions (for controlled trials)

Allocation:

Sequence generation	16a	Method of generating the allocation sequence (eg, computer-generated random numbers), and list of any factors for stratification. To reduce predictability of a random sequence, details of any planned restriction (eg, blocking) should be provided in a separate document that is unavailable to those who enrol participants or assign interventions

Allocation concealment mechanism	16b	Mechanism of implementing the allocation sequence (eg, central telephone; sequentially numbered, opaque, sealed envelopes), describing any steps to conceal the sequence until interventions are assigned
Implementation	16c	Who will generate the allocation sequence, who will enrol participants, and who will assign participants to interventions
Blinding (masking)	17a	Who will be blinded after assignment to interventions (eg, trial participants, care providers, outcome assessors, data analysts), and how
	17b	If blinded, circumstances under which unblinding is permissible, and procedure for revealing a participant's allocated intervention during the trial
**Methods: Data collection, management, and analysis**		
Data collection methods	18a	Plans for assessment and collection of outcome, baseline, and other trial data, including any related processes to promote data quality (eg, duplicate measurements, training of assessors) and a description of study instruments (eg, questionnaires, laboratory tests) along with their reliability and validity, if known. Reference to where data collection forms can be found, if not in the protocol
	18b	Plans to promote participant retention and complete follow-up, including list of any outcome data to be collected for participants who discontinue or deviate from intervention protocols
Data management	19	Plans for data entry, coding, security, and storage, including any related processes to promote data quality (eg, double data entry; range checks for data values). Reference to where details of data management procedures can be found, if not in the protocol
Statistical methods	20a	Statistical methods for analysing primary and secondary outcomes. Reference to where other details of the statistical analysis plan can be found, if not in the protocol
	20b	Methods for any additional analyses (eg, subgroup and adjusted analyses)
	20c	Definition of analysis population relating to protocol non-adherence (eg, as randomised analysis), and any statistical methods to handle missing data (eg, multiple imputation)
**Methods: Monitoring**		
Data monitoring	21a	Composition of data monitoring committee (DMC); summary of its role and reporting structure; statement of whether it is independent from the sponsor and competing interests; and reference to where further details about its charter can be found, if not in the protocol. Alternatively, an explanation of why a DMC is not needed

	21b	Description of any interim analyses and stopping guidelines, including who will have access to these interim results and make the final decision to terminate the trial
Harms	22	Plans for collecting, assessing, reporting, and managing solicited and spontaneously reported adverse events and other unintended effects of trial interventions or trial conduct
Auditing	23	Frequency and procedures for auditing trial conduct, if any, and whether the process will be independent from investigators and the sponsor
**Ethics and dissemination**		
Research ethics approval	24	Plans for seeking research ethics committee/institutional review board (REC/IRB) approval
Protocol amendments	25	Plans for communicating important protocol modifications (eg, changes to eligibility criteria, outcomes, analyses) to relevant parties (eg, investigators, REC/IRBs, trial participants, trial registries, journals, regulators)
Consent or assent	26a	Who will obtain informed consent or assent from potential trial participants or authorised surrogates, and how (see Item 32)
	26b	Additional consent provisions for collection and use of participant data and biological specimens in ancillary studies, if applicable
Confidentiality	27	How personal information about potential and enrolled participants will be collected, shared, and maintained in order to protect confidentiality before, during, and after the trial
Declaration of interests	28	Financial and other competing interests for principal investigators for the overall trial and each study site
Access to data	29	Statement of who will have access to the final trial dataset, and disclosure of contractual agreements that limit such access for investigators
Ancillary and post-trial care	30	Provisions, if any, for ancillary and post-trial care, and for compensation to those who suffer harm from trial participation
Dissemination policy	31a	Plans for investigators and sponsor to communicate trial results to participants, healthcare professionals, the public, and other relevant groups (eg, via publication, reporting in results databases, or other data sharing arrangements), including any publication restrictions
	31b	Authorship eligibility guidelines and any intended use of professional writers
	31c	Plans, if any, for granting public access to the full protocol, participant-level dataset, and statistical code

For peer review

For peer review only - http://bmjopen.bmj.com/site/about/guidelines.xhtml

Appendices

Informed consent materials	32	Model consent form and other related documentation given to participants and authorised surrogates
Biological specimens	33	Plans for collection, laboratory evaluation, and storage of biological specimens for genetic or molecular analysis in the current trial and for future use in ancillary studies, if applicable

*It is strongly recommended that this checklist be read in conjunction with the SPIRIT 2013 Explanation & Elaboration for important clarification on the items. Amendments to the protocol should be tracked and dated. The SPIRIT checklist is copyrighted by the SPIRIT Group under the Creative Commons "Attribution-NonCommercial-NoDerivs 3.0 Unported" license.

For peer review only

BMJ Open

Communicating benign biopsy results by telephone in the NHS Breast Screening Programme: a protocol for a cluster randomised crossover trial

Journal:	*BMJ Open*
Manuscript ID	bmjopen-2018-028679.R1
Article Type:	Protocol
Date Submitted by the Author:	03-May-2019
Complete List of Authors:	Williamson, Sian; University of Warwick, Health Sciences<br>Johnson, Rebecca; University of Warwick,<br>Sandhu, Harbinder; University of Warwick Warwick Medical School, Clinical Trials Unit<br>Parsons, Nicholas; University of Warwick, Warwick Medical School<br>Jenkins, Jacquie; Public Health England, National Programme Manager – NHS Breast Screening Programme<br>Casey, Margaret; Royal Wolverhampton NHS Trust, Clinical Nurse Specialist Breast Care<br>Kearins, Olive; Public Health England, National Lead Breast Screening QA<br>Taylor-Phillips, Sian; University of Warwick, Warwick Medical School
**Primary Subject Heading**:	Health services research
Secondary Subject Heading:	Communication, Oncology, Public health
Keywords:	PREVENTIVE MEDICINE, Health policy < HEALTH SERVICES ADMINISTRATION & MANAGEMENT, Protocols & guidelines < HEALTH SERVICES ADMINISTRATION & MANAGEMENT, Quality in health care < HEALTH SERVICES ADMINISTRATION & MANAGEMENT

SCHOLARONE™
Manuscripts

Protocol paper V.7 01.05.2019

Communicating benign biopsy results by telephone in the NHS Breast Screening Programme: a protocol for a cluster randomised crossover trial

Sian Williamson, s.williamson@warwick.ac.uk (PhD Student, University of Warwick),

Rebecca Johnson, rebecca.johnson@warwick.ac.uk (Senior Research Fellow, University of Warwick),

Harbinder Sandhu, harbinder.k.sandhu@warwick.ac.uk (Associate Professor, University of Warwick),

Nick Parsons, nick.parsons@warwick.ac.uk (Statistician, Principal Research Fellow, University of Warwick)

Jacquie Jenkins, jacquie.jenkins@phe.gov.uk (National Programme Manager – NHS Breast Screening Programme),

Margaret Casey, margaret.casey@nhs.net (Clinical Nurse Specialist Breast Care),

Olive Kearins, Olive.Kearins@phe.gov.uk (National Lead Breast Screening QA, Public Health England)

& Sian Taylor-Phillips, s.taylor-phillips@warwick.ac.uk (Associate Professor, University of Warwick)

Corresponding author: Sian Taylor-Phillips

Word count (excluding abstract): 3,753 words

Keywords: Mass screening, communication, cancer screening, RCT

For peer review only - http://bmjopen.bmj.com/site/about/guidelines.xhtml

Protocol paper V.7 01.05.2019

Abstract

Introduction – One of the main harms from breast cancer screening is the anxiety caused by false positive results. Various factors may be associated with false-positive anxiety. One modifiable factor may be the method of communication used to deliver results. The aim of this study is to measure the effect on anxiety of receiving benign biopsy results in-person or by telephone.

Methods and analysis – This is a multi-centre cluster randomised crossover trial in the English NHS Breast Screening Programme (NHSBSP) involving repeated survey measures at four time points. Participants will be women of screening age who have a biopsy following a suspicious mammography result, who ultimately receive a benign or normal (B1) result.

Centres will trial both telephone and in-person results on a month-by-month basis, being randomised to which communication method will be trialled first. Women will be blinded to the method of communication they will receive.

The analysis will compare women who have received telephone results and women who have received in-person results. The primary outcome measure will be anxiety (measured by the Psychological Consequences Questionnaire) after receiving results, whilst controlling for baseline anxiety. Secondary outcome measures will include anxiety at 3 and 6 months post-results, understanding of results and patient preferences for how results are communicated. Qualitative telephone interviews will also be conducted to further explore women's reasons for communication preferences. Qualitative and quantitative data will be integrated after initial separate analysis using the Pillar Integration Process.

Ethics and dissemination – This study has been approved by the Public Health England Breast Screening Programme Research Advisory Committee, (BSPRAC_0013, ODR1718_040) and the HRA NHS West Midlands - Coventry & Warwickshire Research Ethics Committee (17/WM/0313). The findings from this study will be disseminated to key stakeholders within the NHSBSP and via academic publications.

Registration details – ISRCTN36997684 https://doi.org/10.1186/ISRCTN36997684

For peer review only

Protocol paper V.7 01.05.2019

Trial sponsor – This research is part of a PhD award and is funded by the Economic and Social Research Council (ESRC) Doctoral Training Centre at the University of Warwick and Public Health England. The sponsor for this research is Jane Prewett (sponsorship@warwick.ac.uk).

Article summary

Strengths and limitations of this study

- This is the first cluster randomised crossover trial to measure the impact of communication method on patient outcomes when delivering results in breast screening
- The mixed-methods design adds depth to our understanding of communication preferences and the mechanisms by which anxiety may be affected
- The study is in English breast screening centres and generalisability to other contexts should be carefully considered

For peer review only

Protocol paper V.7 01.05.2019

Introduction

The UK National Health Service Breast Screening Programme (NHSBSP) is a population-based screening programme that aims to detect early signs of breast cancer. Asymptomatic women aged 50-70 are invited to attend every three years for a mammogram. The results of the mammogram take a maximum of two weeks as per NHSBSP guidelines (1). If a suspected abnormality is found on the mammogram then the woman is invited back to the clinic for follow-up tests, usually within the next 2 weeks.

Follow-up tests can include clinical examination, a further mammogram or ultrasound. If these indicate suspicion of cancer then the woman receives a core needle biopsy, involving the removal of sections of tissue from the suspicious breast region. This tissue can then be pathologically analysed. Although the procedure is designed to be minimally invasive, leaving only minor bruising, some women find the experience painful and distressing. It is standard practice to perform all follow-up tests (clinical exam, mammogram, ultrasound and biopsy) on the same day in an assessment clinic to avoid unnecessary extra waiting time for patients. Results of diagnostic tests are discussed on a case-by-case basis at multidisciplinary team meetings (MDT) using a triple-assessment of clinical examination, imaging and biopsy report. Clinical guidelines recommend that all follow-up results should be delivered to the woman within one week (2). These results are delivered by either telephone or in-person, depending on the procedure at the breast screening centre.

For peer review only

Screening programmes should provide benefit that outweighs both physical and psychological harm (3, 4). One of the main harms from breast screening is the anxiety caused by false positive results (5). A false-positive result is when a woman has been identified at the screening phase as potentially having cancer, but follow up tests have revealed no abnormalities. Receiving a false-positive result from screening is very common, with the majority of women who are recalled ultimately being given a false-positive result (6). In England, around half of women receive the all clear results at the time of the follow-up tests. Women who are invited to be screened have no symptoms of breast cancer at the time of their initial mammogram. Telling a woman that something suspicious has been found in the mammogram and that further tests are needed can make her feel very anxious and believe that she

might have cancer (7). For some women, once results confirm the absence of cancer, anxiety declines. However, anxiety can remain elevated for much longer, lasting up to three years after receiving the benign result and leading into the next screening invitation (8). This is an issue, as the NHSBSP has a duty to minimize the harm caused by screening.

There are various factors that may be associated with heightened anxiety during screening such as family history, lower education, younger age and individual differences in personality (1, 9-13). These factors are unmodifiable and tend to be focused at the level of the individual, which makes the minimisation of screening anxiety a challenge. However, it is possible that there are modifiable changes that can be made at the organisational level of screening that may minimise the impact of anxiety. One of these changes is the method of communication used to deliver results.

NHSBSP guidelines for communicating results state that telephone results ‘should not be routinely offered’ (14). However, most breast screening centres in the UK deliver benign results to the woman over the telephone. Some breast care nurses remain concerned about how the communication method used to deliver results may contribute to the anxiety experienced by women attending screening (2). Telephone results may offer advantages, eliminating the stress and costs associated with transport, parking and anxiously waiting in a clinic for results. However, telephone results eliminates the in-person encounter, meaning all communication is verbal only. Research from other areas has shown that non-verbal communication plays a key role in enhancing understanding and minimising anxiety (15).

The communication methods used to deliver benign results in the NHSBSP has not yet been explored. Therefore, the impact of this communication on women receiving a benign result is unknown.

Aim of the study

The aim of this study is to compare anxiety in women receiving benign biopsy results from the NHSBSP via telephone results or in-person.

For peer review only - http://bmjopen.bmj.com/site/about/guidelines.xhtml

Protocol paper V.7 01.05.2019

Methods and analysis

Study design

Patient and public involvement from the charity Independent Cancer Patient Voices was used to guide the design of the study to ensure the acceptability of the method and appropriateness of the participant materials.

Participants and settings

The study design chosen was a multi-centre cluster randomised crossover trial. The randomisation allows for the direct comparison of study outcomes between women who received telephone results and women who received in-person results, whilst controlling for confounding factors such as education, age and individual differences in baseline anxiety.

This trial will be conducted in a breast screening centre setting. The study will take place in the UK where women are invited to attend breast screening every 3 years for digital mammography. The study will take place across four time points (see Table 1) with a survey completed by participants at each stage. The study participants will be recruited from 4 English Breast Screening centres across different regions.

Participants will be women between the ages of 47-73 attending the NHSBSP for further tests following a suspicious mammogram. This includes women offered routine screening between ages 50-70 and those receiving extra rounds of screening between the ages of 47-49 or 71-73 as part of the UK age extension trial (16). Women will be recruited at the assessment clinic pre-biopsy. However, only women who have receive a benign (B2) or normal (B1) biopsy result will be included in the longitudinal data collection. Participants will not be included in the study if they presented symptomatically to the breast clinic, if they are not the recommended screening age, if they do not receive a biopsy, if they do not have English as a first or second language and if they do not have the capacity to consent.

For peer review only - http://bmjopen.bmj.com/site/about/guidelines.xhtml

Protocol paper V.7 01.05.2019

Table 1: Time points for the study

Time point	Sample	Survey content	Method
Time 1 – at assessment clinic	Women attending assessment follow-up clinic	Demographic information<br>Communication preferences<br>Baseline anxiety score (PCQ)<br>Contact details	In-person
Time 2 – after receiving results	Women from time 1 who had a normal or benign biopsy	Communication preferences<br>Repeat anxiety score (PCQ)<br>Measure of understanding	By post
Time 3 – Three month follow-up	Women from time 1 who had a normal or benign biopsy	Repeat anxiety score (PCQ)	By post
Time 4 – Six month follow-up	Women from time 1 who had a normal or benign biopsy	Repeat anxiety score (PCQ)	By post

<u>Measuring anxiety</u>

The Psychological Consequences Questionnaire (PCQ) was selected as the most appropriate measure of anxiety in the breast screening setting (17) and was embedded in the participant surveys (see Appendix 1). The PCQ is a disease specific measure, focusing on breast cancer specific anxiety across 12 questions on three dimensions: emotional, social and physical anxiety. The disease specific measure avoids the contrast-validity that is associated with using generic anxiety measures in the breast screening context (18). The PCQ is widely used in the breast screening setting (1, 8, 19-21).

Participants will rate their anxiety on the PCQ by judging each statement on a scale of 0 (not at all) to 3 (quite a lot of the time). Women will be asked "*<u>Over the last week</u>* how often have you experiences the following things because of *<u>thoughts and feelings about breast cancer</u>*:"

For peer review only - http://bmjopen.bmj.com/site/about/guidelines.xhtml

For peer review only

Randomisation and blinding

Each centre will be randomised to one of two intervention arms by computer generated random numbers from the trial team base at the University of Warwick (see Table 2). The arm relates to whether the first month at each centre will be telephone or in-person results. Each centre will commence with the communication method randomised in month 1 and continue to alternate between the two communication methods for the duration of the study. This approach allowed for each centre to use both methods of communication, controlling for previous experience (e.g. centres who already telephone are more experienced and therefore women receiving telephone results from this centre might be less anxious).

This approach was selected instead of a block approach, where each centre would deliver 6 months in-person followed by 6 months of telephone. The reason for this was to avoid potential bias from unforeseen centre drop out.

Table 2: Allocation of communication method by arm of the trial

Month	Arm 1	Arm 2
1	Telephone	In-person
2	In-person	Telephone
3	Telephone	In-person
4	In-person	Telephone
5	Telephone	In-person
6	In-person	Telephone

This design was chosen to ensure balance between trial arms at each site whilst addressing practical constraints. Balance between trial arms at each site is important to account for centre level confounders such as staff communication skills and centre level processes.

Individual randomisation was not possible as it interfered with screening centres workflow and fail-safe mechanisms to ensure that every woman was contacted with the correct information within the correct time frame.

Women will be allocated to receive their result based on the date of their attendance at the assessment visit. However, participants will be blinded to the randomisation

For peer review only - http://bmjopen.bmj.com/site/about/guidelines.xhtml

month. Breast screening staff will not be blinded, as they will be delivering the results and scheduling the appointments.

Allocation of communication method

There are two types of centres who may be involved in the research: centres who currently deliver benign results in-person and centres who currently deliver benign results by telephone.

For centres currently delivering results in-person, the following process for scheduling a results appointment will be observed: During in-person study months, all consenting women at time 1 will be given an appointment to re-attend to receive their results in-person. During telephone study months, all consenting women at time 1 will be given an appointment to re-attend to receive their results in person. However, they will consent to potentially receiving results over the telephone, which will happen prior to their scheduled appointment. Only benign women will be telephoned, with all women receiving other results attending their scheduled appointment

For centres currently delivering results by telephone, the following process for scheduling a results appointment will be observed: All consenting women will be informed that, when their results are ready, they will be contacted by telephone to arrange an appointment to come back for their results. During the ‘telephone’ months of the study, women will be telephoned as expected. However, instead of arranging an appointment during this telephone call, results will be delivered. This means that, for women who go on to have a cancer result, they can be telephoned to arrange an appointment to attend in-person, as expected. This is in-line with standard practice at these centres. During the in-person months, all consenting women will be informed that, when their results are ready, they will be telephoned to arrange an appointment to come back for their results. These women will be telephoned to arrange an appointment to come back in-person, and at that appointment they will receive their benign/normal result.

All women not enrolled in the study will receive their screening result based on the current standard practice at the attended centre.

For peer review only - http://bmjopen.bmj.com/site/about/guidelines.xhtml

Data collection

Time point 1

Participant recruitment at time one will occur concurrently at each breast screening centre.

Women will be approached during their assessment visit by breast care nurses with Good Clinical Practice (GCP) training. These women will have been recalled from a previous mammogram and may have a biopsy as part of the assessment clinic. The study will be explained to potential participants and nurses will go through the informed consent process. Consenting women will fill out the Time point 1 survey with study responses collected and stored securely before the participant leaves the assessment clinic.

Multi-disciplinary team meeting

At the local level multi-disciplinary team meeting for breast screening staff, women recruited into the study will be included in further time points if they receive a benign (B2) or normal (B1) result. A breast care review will compile the contact details of eligible women into a spreadsheet to be sent securely to the research team.

Time point 2

The research team will distribute Time point 2 surveys to eligible women with a pre-paid return envelope. If no response is received within a week, the research team will contact women by telephone as a reminder. A maximum of two telephone contact attempts will be made. This is to ensure anxiety and understanding are measured at the crucial post-results stage of the screening process.

As part of Time point 2 survey, women will be asked if they would like to participate in further research involving an interview about their experience of receiving a screening result.

For peer review only - http://bmjopen.bmj.com/site/about/guidelines.xhtml

Time point 3

The research team will distribute Time point 3 surveys, three months after the biopsy result was received.

Time point 4

The research team will distribute Time point 4 surveys, six months after the biopsy result was received.

Qualitative telephone interviews

The qualitative telephone interviews will explore why women prefer certain methods of communication. Women will be recruited from the time point 2 survey. Women who express an interest in participating will be sent further information about the interviews. If they wish to participate, women will return the consent form in the pre-paid envelope. Women will then be contacted by telephone by the research team to be interviewed.

The semi-structured telephone interviews will each take 10-20 minutes. Questions will encompass the woman's experience of receiving a result from screening. This will involve asking how the woman felt, whether she understood her result and an exploration of her views on different methods for communicating results. Interviews will be audio-recorded and transcribed verbatim. Data collection will cease once no further themes emerge and data saturation is reached.

Mixed methods integration

Using a mixed methods approach, the quantitative preference survey data will be combined with the findings from the qualitative interviews. The quantitative data asks a binary choice question regarding women's communication preferences (telephone or in-person), whilst the qualitative interviews expand on this by exploring how women justify certain preferences for communication. In mixing the data, the qualitative data will be used to *expand* the understanding of the findings from the quantitative surveys (22). Expansion provides richness and detail, expanding why and how women form communication preferences and moving understanding of preferences *beyond* what quantitative data or qualitative data in isolation can

For peer review only - http://bmjopen.bmj.com/site/about/guidelines.xhtml

elucidate. The value added by integrating the knowledge of both *what* women prefer and *why* is in the completeness of our understanding of preferences, making the evidence that will inform the NHSBSP policy decisions comprehensive and patient-centred.

For peer review only

Sample size considerations

In order to determine the sample size for a clustered randomised cross-over study, a full specification of the important within-cluster (centre) between-period and within-period correlations is required (23). There is currently no available evidence on the magnitude of these correlations in our selected setting, so rather than arbitrarily selecting values we adopt a conservative approach and assume that the (within-centre) between-period correlations are zero and proceed to power as if the design were a cluster randomised design. Although the cross-over aspect of the design is not explicitly accounted for in the sample size calculation, it is incorporated fully in the analysis of the primary study outcome (PCQ anxiety score at time-point 2). The aim of the study to be able to detect a clinically significant difference of 3 points in the PCQ (the difference between the score on one statement being 0, not at all, and 3, quite a lot of the time).

[Insert Figure 1 here]

Figure 1: Flow of required sample size for the study

Assuming the primary outcome is approximately normally distributed, and the test is at the 5% significance level with 80% power to detect an effect of the specified size, 194 participants are required at time 2 i.e. 97 participants per arm. Allowing for attrition rate between time point 1 and time point 2 due to participant withdrawal (15%) and participant eligibility (50%), a total of 457 participants will be recruited at time point 1. Participant withdrawal was calculated based upon a mean response rate of 60% from previous research using postal surveys in a medical setting (24) with a loss of 15% at each time point as a conservative estimate.

In order to account for clustering due to the recruiting centre, design effect was applied that inflated the sample size. The intra-cluster (within-centre) correlation coefficient (ICC) was set to be 0.01 and the number of observations within each cluster were assumed to be equal. With the sample size of 194 women, divided by the number of centres (4) and then divided by the number of interventions (2), this led to the number of observations within a cluster being 48.2. This gave a design

For peer review only - http://bmjopen.bmj.com/site/about/guidelines.xhtml

effect of 1.49. Taking this into account, the sample size needed to achieve statistical significance (194 women) was multiplied by the design effect, giving a total sample of 290 women at time point 2 when rounded up (see Figure 1).

31,926 women are recalled for a biopsy each year. If half of the 31,926 biopsies come back as benign, this leaves a potential sample of 15,963. This means that, on average across 80 breast screening centres, each centre will have around 200 benign biopsy results each year. Therefore, assuming 50% participation rates of eligible women, recruitment will require four centres for one year.

For peer review only

Outcomes and study measures (primary outcome, secondary outcomes)

Primary outcome

The primary outcome is the PCQ anxiety score at time point 2.

A comparison in anxiety score will be made between women who receive results in-person and women who receive results over the telephone.

Secondary outcomes

Secondary outcomes are:

- PCQ anxiety score at 3 month follow-up (time 3)
- PCQ anxiety score at 6 month follow-up (time 4)
- Subjective understanding of results (time 2)
    - Measured using a survey question designed in collaboration with NHSBSP stakeholders
- Objective understanding of results (time 2)
    - Measured using a survey question designed in collaboration with NHSBSP stakeholders
- Quantitative preferences for results communication before results (time 1)
- Quantitative preferences for results communication after results (time 2)
- Qualitative preferences for results communication before results (time 1)
- Qualitative preferences for results communication after results (time 2)

For peer review only - http://bmjopen.bmj.com/site/about/guidelines.xhtml

Protocol paper V.7 01.05.2019

Analysis

Quantitative data - Statistical analysis

A formal and more detailed statistical analysis plan (SAP) will be developed by the trial team prior to the completion of recruitment.

Primary outcome

The primary analysis will use a mixed effects linear regression model to estimate the effects of communication method on anxiety (time 2), after adjusting for baseline anxiety (time 1). PCQ score will be treated as a continuous variable and to be approximately normally distributed for all analyses.

The model set-up and fixed and random effects are as follows:

- Response variable – Anxiety at time 2 (PCQ)
- Baseline – Anxiety at time 1 (PCQ)
- Fixed explanatory effects (model covariates) – age, ethnicity, previous attendance, previous biopsy, education, marital status.
- Random effects – centre and temporal (period) effects
- Comparator variable - Method of communication received – telephone or in-person

Statistical significance will be assessed at the 5% level.

Secondary outcomes

Longitudinal anxiety scores at 3 and 6 months will be analysed in the same way as the primary anxiety outcome.

Differences in understanding score between communication methods groups will be assessed using a logistic regression model, adjusting for fixed effects.

- Outcome variable – Subjective understanding score (binary – yes or no), objective understanding score (binary – right or wrong)
- Fixed effects – age, ethnicity, previous attendance, previous biopsy, education, marital status.
- Comparator variable - Method of communication received (telephone/in-person)

For peer review only - http://bmjopen.bmj.com/site/about/guidelines.xhtml

Preference data from the quantitative surveys will be presented in the form of percentages. All analyses will be implemented using IBM SPSS Statistics 25.

Qualitative data analysis

Qualitative preference data from the time point 1 and time point 2 surveys and data from the telephone interviews will be analysed using inductive thematic analysis (25), managed using NVivo10.

Mixed-methods integration

To integrate the quantitative and qualitative preference data, the Pillar Integration Process will be used (26). This analytical integration technique uses four systematic stages (listing, matching, checking and pillar-building) to identify and examine connections and discrepancies in qualitative and quantitative findings. It allows for the visual display of the data and findings; this enhances overall transparency of the integration approach and the results of such an integration.

For peer review only

Protocol paper V.7 01.05.2019

Ethics

Attention was given to the various ethical challenges of the trial. The main ethical issue will be the use of sensitive patient information (addresses, telephone numbers). Participants will be told explicitly how their contact details will be used and stored throughout the data collection process. Participants will give informed consent for their contact details to be used for the purposes of the study.

All electronic data will be transferred securely in a password protected excel document from secure email accounts. All raw survey data will be collected directly from the centres by the lead researcher and be transferred in a secure lock-box.

Ethical approval for this study was granted by the following organisations:

- Public Health England Breast Screening Programme Research Advisory Committee, BSPRAC_0013 (ODR1718_040)
- HRA NHS West Midlands - Coventry & Warwickshire Research Ethics Committee (17/WM/0313)

Dissemination

Results from the trial will disseminated directly to key stakeholders within the NHSBSP. This will encourage discussion regarding how benign results are communicated in breast screening, and how this might best be implemented in order to minimise the anxiety women experience.

The results will also be disseminated via academic publications.

For peer review only

Protocol paper V.7 01.05.2019

Current study status

The trial began recruitment in February 2018. Data collection is due to conclude in March 2019. The trial statisticians (SW and NP) have received recruitment data but no results will be transferred to the statisticians until recruitment is closed in March 2019.

Author contributions

SZW is the lead researcher. SZW and STP drafted the manuscript. SZW, RJ, HS and STP participated in the design of the study. JJ, MC and OK are key stakeholders in the NHSBSP who assisted with the study design. NP aided with the statistical analysis. All authors have reviewed and approved the manuscript.

Funding statement

This research is part of a PhD award and is funded by the Economic and Social Research Council (ESRC) Doctoral Training Centre at the University of Warwick. The funding has been awarded for this studentship to Sian Williamson for her PhD project for 4 years of full-time study. The award consists of payment of academic fees and a maintenance award.

A further contract between the University of Warwick, Public Health England (PHE) and the PhD Student (Sian Williamson) has secured £4,000 in research expenses.

Competing interests

None declared

For peer review only

Protocol paper V.7 01.05.2019

References

1. Brett J, Bankhead C, Henderson B, Watson E, Austoker J. The psychological impact of mammographic screening. A systematic review. Psycho-Oncology. 2005;14(11):917-38.
2. Chapman K. Interim quality assurance guidelines for clinical nurse specialists in breast cancer screening. 2012.
3. Raffle AE, Gray JM. Screening: evidence and practice: Oxford University Press; 2007.
4. Marmot MG, Altman D, Cameron D, Dewar J, Thompson S, Wilcox MJBjoc. The benefits and harms of breast cancer screening: an independent review. 2013;108(11):2205.
5. Bond M, Pavey T, Welch K, Cooper C, Garside R, Dean S, et al. Systematic review of the psychological consequences of false-positive screening mammograms. 2013.
6. DeFrank JT, Brewer N. A model of the influence of false-positive mammography screening results on subsequent screening. Health psychology review. 2010;4(2):112-27.
7. Solbjør M, Forsmo S, Skolbekken J-A, Sætnan AR. Experiences of recall after mammography screening—a qualitative study. Health care for women international. 2011;32(11):1009-27.
8. Brett J, Austoker J. Women who are recalled for further investigation for breast screening: psychological consequences 3 years after recall and factors affecting re-attendance. Journal of Public Health. 2001;23(4):292-300.
9. Matteau TM. Psychology and screening: narrowing the gap between efficacy and effectiveness. British Journal of Clinical Psychology. 1994;33(1):1-10.
10. Ando N, Iwamitsu Y, Kuranami M, Okazaki S, Nakatani Y, Yamamoto K, et al. Predictors of psychological distress after diagnosis in breast cancer patients and patients with benign breast problems. Psychosomatics. 2011;52(1):56-64.
11. Keyzer-Dekker CM, de Vries J, Mertens MC, Roukema JA, van der Steeg AF. Cancer or no cancer: the influence of trait anxiety and diagnosis on quality of life with breast cancer and benign disease: a prospective, longitudinal study. World journal of surgery. 2013;37(9):2140-7.
12. Montgomery M, McCrone SH. Psychological distress associated with the diagnostic phase for suspected breast cancer: systematic review. Journal of advanced nursing. 2010;66(11):2372-90.
13. Sweeny K, Andrews SE. Mapping individual differences in the experience of a waiting period. Journal of personality and social psychology. 2014;106(6):1015.
14. England. PH. NHS public health functions agreement 2017- 18 Service specification no.24 Breast Screening Programme. In: England. PH, editor. 2017.
15. Knapp ML, Hall J. Nonverbal behavior in human interaction. Wadsworth: New York; 2002.
16. 2000. CgIBMNLoMUF. Identifier NCT01081288; Extending the National Health Service (NHS) Breast Screening Age Range (AgeX). 5 March 2010 (16 November 2018).
17. Cockburn J, De Luise T, Hurley S, Clover K. Development and validation of the PCQ: a questionnaire to measure the psychological consequences of screening mammography. Social science & medicine. 1992;34(10):1129-34.

For peer review only - http://bmjopen.bmj.com/site/about/guidelines.xhtml

18. Brodersen J, Thorsen H, Cockburn J. The adequacy of measurement of short and long-term consequences of false-positive screening mammography. Journal of medical screening. 2004;11(1):39-44.
19. Sweeny K, Christianson D, McNeill J. The Psychological Experience of Awaiting Breast Diagnosis. 2018.
20. Cockburn J, Staples M, Hurley SF, De Luise T. Psychological consequences of screening mammography. Journal of Medical Screening. 1994;1(1):7-12.
21. Brewer NT, Salz T, Lillie SE. Systematic Review: The Long-Term Effects of False-Positive MammogramsThe Long-Term Effects of False-Positive Mammograms. Annals of internal medicine. 2007;146(7):502-10.
22. Creswell JW, Klassen AC, Plano Clark VL, Smith KC. Best practices for mixed methods research in the health sciences. Bethesda (Maryland): National Institutes of Health. 2011:2094-103.
23. Arnup SJ, McKenzie JE, Hemming K, Pilcher D, Forbes ABJT. Understanding the cluster randomised crossover design: a graphical illustration of the components of variation and a sample size tutorial. 2017;18(1):381.
24. Asch DA, Jedrziewski MK, Christakis NAJJoce. Response rates to mail surveys published in medical journals. 1997;50(10):1129-36.
25. Braun V, Clarke VJQrip. Using thematic analysis in psychology. 2006;3(2):77-101.
26. Johnson RE, Grove AL, Clarke AJJoMMR. Pillar Integration Process: A Joint Display Technique to Integrate Data in Mixed Methods Research. 2017:1558689817743108.

For peer review only

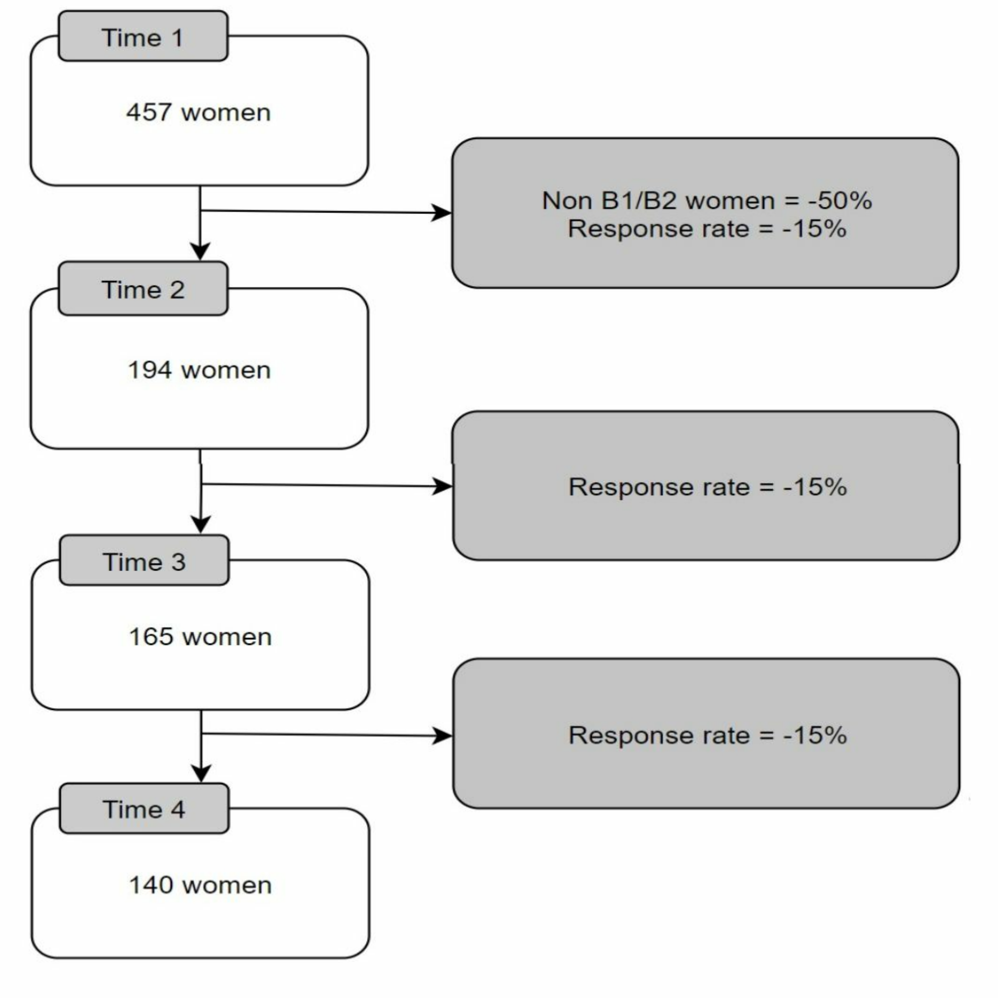

Figure 1 - Flow of required sample size for the study

89x89mm (300 x 300 DPI)

Survey (time 1) – V.6 05.04.2018

Communicating results in breast screening: Research survey – Time 1

Public Health England

WARWICK
THE UNIVERSITY OF WARWICK

NHS
Cancer Screening Programmes

PARTICIPANT NAME: ______________________________

(Please ensure this is completed)

For researcher use only:

Participant ID number: ______________________________

B1 or B2 result:

- ☐ Yes
- ☐ No

For peer review only - http://bmjopen.bmj.com/site/about/guidelines.xhtml

Survey (time 1) – V.6 05.04.2018

Participant information

1) What is your age?

___________ years

2) Have you attended screening before?

☐ Yes
☐ No

2a) If yes, did you have a biopsy (a needle inserted into the breast to remove tissue)?

☐ Yes
☐ No
☐ Can't remember

3) Please tick the highest level of education you have achieved:

☐ No schooling completed
☐ High school or equivalent
☐ Some college
☐ Bachelor's degree
☐ Master's degree
☐ Doctoral degree
☐ Professional degree
☐ Other

4) Please tick your marital status:

☐ Single
☐ Domestic partnership
☐ Married
☐ Divorced
☐ Widowed
☐ Separated

5) What is your ethnic group?

White:

☐ English/Welsh/Scottish/Northern Irish/British
☐ Irish
☐ Gypsy or Irish Traveller
☐ Any other White background

Asian/Asian British:

☐ Indian
☐ Pakistani
☐ Bangladeshi
☐ Chinese
☐ Any other Asian background

Mixed/multiple ethnic groups:

☐ White and Black Caribbean
☐ White and Black African
☐ White and Asian
☐ Any other Mixed/Multiple ethnic background

Black/African/Caribbean/Black British:

☐ African
☐ Caribbean
☐ Any other Black/African/Caribbean background

Other ethnic group:

☐ Arab
☐ Any other ethnic group

For peer review only

Preferences for communication

When a screening result is not cancer, some centres deliver these results in-person and some deliver these results over the telephone. We want to know how you would prefer to receive screening results, *if you were given the choice.*

Please note, if you tick a preference, you may not necessarily get your result delivered this way.

6) How would you prefer to receive your results from screening?

☐ In-person at the screening centre
☐ By telephone

7) Why would you prefer to receive your results this way?

Please write a brief explanation:

For peer review only

For peer review only - http://bmjopen.bmj.com/site/about/guidelines.xhtml

Survey (time 1) – V.6 05.04.2018

How do you feel?

8) *Over the last week* how often have you experiences the following things because of *thoughts and feelings about breast cancer*:

	Not at all	Rarely	Some of the time	Quite a lot of the time
Had trouble sleeping	0	1	2	3
Experienced a change in appetite	0	1	2	3
Been unhappy or depressed	0	1	2	3
Been scared and panicky	0	1	2	3
Felt nervous or strung up	0	1	2	3
Felt under strain	0	1	2	3
Found you have been keeping things from those who are close to you	0	1	2	3
Found yourself taking things out on other people	0	1	2	3
Found yourself noticeably withdrawing from those who are close to you	0	1	2	3
Had difficultly doing things around the house that you normally do	0	1	2	3
Had difficulty meeting work or other commitments	0	1	2	3
Felt worried about your future	0	1	2	3

For peer review only - http://bmjopen.bmj.com/site/about/guidelines.xhtml

Survey (time 1) – V.6 05.04.2018

9) Please provide a contact name and address for follow-up surveys to be sent:

(Please note that this address will remain anonymous and all contact information will be destroyed once the study is complete. This address will only be used for the purposes of this research to post follow-up surveys.)

____

____

____

____

____

10) Please provide a contact telephone number:

(Please note that this contact number will remain anonymous and all contact information will be destroyed once the study is complete. This telephone number will only be used for the purposes of this research to remind you of the first follow-up survey.)

____

11) Would you like to hear about the results from this research?

(If you choose yes, you will be sent a copy of the results from this research via the address provided)

☐ Yes

☐ No

For peer review only - http://bmjopen.bmj.com/site/about/guidelines.xhtml
For peer review only

STANDARD PROTOCOL ITEMS: RECOMMENDATIONS FOR INTERVENTIONAL TRIALS

SPIRIT 2013 Checklist: Recommended items to address in a clinical trial protocol and related documents*

Section/item	Item No	Description
Title<br>**Page 1**	1	Descriptive title identifying the study design, population, interventions, and, if applicable, trial acronym
Trial registration<br>**Page 2**	2a	Trial identifier and registry name. If not yet registered, name of intended registry
**n/a**	2b	All items from the World Health Organization Trial Registration Data Set
Protocol version<br>***Top of page***	3	Date and version identifier
Funding<br>**Page 18**	4	Sources and types of financial, material, and other support
Roles and responsibilities<br>**Page 18**	5a	Names, affiliations, and roles of protocol contributors
**Page 3**	5b	Name and contact information for the trial sponsor
**n/a - No role in these activities**	5c	Role of study sponsor and funders, if any, in study design; collection, management, analysis, and interpretation of data; writing of the report; and the decision to submit the report for publication, including whether they will have ultimate authority over any of these activities
**n/a**	5d	Composition, roles, and responsibilities of the coordinating centre, steering committee, endpoint adjudication committee, data management team, and other individuals or groups overseeing the trial, if applicable (see Item 21a for data monitoring committee)

For peer review only - http://bmjopen.bmj.com/site/about/guidelines.xhtml

Introduction

Background and rationale **Page 4**	6a	Description of research question and justification for undertaking the trial, including summary of relevant studies (published and unpublished) examining benefits and harms for each intervention
**n/a**	6b	Explanation for choice of comparators
Objectives **Page 6**	7	Specific objectives or hypotheses
Trial design **Page 6**	8	Description of trial design including type of trial (eg, parallel group, crossover, factorial, single group), allocation ratio, and framework (eg, superiority, equivalence, noninferiority, exploratory)

Methods: Participants, interventions, and outcomes

Study setting **Page 6**	9	Description of study settings (eg, community clinic, academic hospital) and list of countries where data will be collected. Reference to where list of study sites can be obtained
Eligibility criteria **Page 6**	10	Inclusion and exclusion criteria for participants. If applicable, eligibility criteria for study centres and individuals who will perform the interventions (eg, surgeons, psychotherapists)
Interventions **Page 8**	11a	Interventions for each group with sufficient detail to allow replication, including how and when they will be administered
**n/a**	11b	Criteria for discontinuing or modifying allocated interventions for a given trial participant (eg, drug dose change in response to harms, participant request, or improving/worsening disease)
**n/a**	11c	Strategies to improve adherence to intervention protocols, and any procedures for monitoring adherence (eg, drug tablet return, laboratory tests)
**n/a**	11d	Relevant concomitant care and interventions that are permitted or prohibited during the trial

For peer review only - http://bmjopen.bmj.com/site/about/guidelines.xhtml

Outcomes **Page 14**	12	Primary, secondary, and other outcomes, including the specific measurement variable (eg, systolic blood pressure), analysis metric (eg, change from baseline, final value, time to event), method of aggregation (eg, median, proportion), and time point for each outcome. Explanation of the clinical relevance of chosen efficacy and harm outcomes is strongly recommended
Participant timeline **Page 10**	13	Time schedule of enrolment, interventions (including any run-ins and washouts), assessments, and visits for participants. A schematic diagram is highly recommended (see Figure)
Sample size **Page 13**	14	Estimated number of participants needed to achieve study objectives and how it was determined, including clinical and statistical assumptions supporting any sample size calculations
Recruitment **Page 13**	15	Strategies for achieving adequate participant enrolment to reach target sample size
**Methods: Assignment of interventions (for controlled trials)**		
Allocation:		
Sequence generation **Page 8**	16a	Method of generating the allocation sequence (eg, computer-generated random numbers), and list of any factors for stratification. To reduce predictability of a random sequence, details of any planned restriction (eg, blocking) should be provided in a separate document that is unavailable to those who enrol participants or assign interventions
Allocation concealment mechanism **n/a**	16b	Mechanism of implementing the allocation sequence (eg, central telephone; sequentially numbered, opaque, sealed envelopes), describing any steps to conceal the sequence until interventions are assigned
Implementation **Page 8**	16c	Who will generate the allocation sequence, who will enrol participants, and who will assign participants to interventions
Blinding (masking) **Page 8**	17a	Who will be blinded after assignment to interventions (eg, trial participants, care providers, outcome assessors, data analysts), and how
**n/a**	17b	If blinded, circumstances under which unblinding is permissible, and procedure for revealing a participant's allocated intervention during the trial

For peer review only - http://bmjopen.bmj.com/site/about/guidelines.xhtml

Methods: Data collection, management, and analysis

Data collection methods **Page 15**	18a	Plans for assessment and collection of outcome, baseline, and other trial data, including any related processes to promote data quality (eg, duplicate measurements, training of assessors) and a description of study instruments (eg, questionnaires, laboratory tests) along with their reliability and validity, if known. Reference to where data collection forms can be found, if not in the protocol
**n/a**	18b	Plans to promote participant retention and complete follow-up, including list of any outcome data to be collected for participants who discontinue or deviate from intervention protocols
Data management **n/a**	19	Plans for data entry, coding, security, and storage, including any related processes to promote data quality (eg, double data entry; range checks for data values). Reference to where details of data management procedures can be found, if not in the protocol
Statistical methods **Page 15**	20a	Statistical methods for analysing primary and secondary outcomes. Reference to where other details of the statistical analysis plan can be found, if not in the protocol
**Page 15**	20b	Methods for any additional analyses (eg, subgroup and adjusted analyses)
**n/a**	20c	Definition of analysis population relating to protocol non-adherence (eg, as randomised analysis), and any statistical methods to handle missing data (eg, multiple imputation)

Methods: Monitoring

Data monitoring **n/a**	21a	Composition of data monitoring committee (DMC); summary of its role and reporting structure; statement of whether it is independent from the sponsor and competing interests; and reference to where further details about its charter can be found, if not in the protocol. Alternatively, an explanation of why a DMC is not needed
**n/a**	21b	Description of any interim analyses and stopping guidelines, including who will have access to these interim results and make the final decision to terminate the trial
Harms **n/a**	22	Plans for collecting, assessing, reporting, and managing solicited and spontaneously reported adverse events and other unintended effects of trial interventions or trial conduct
Auditing **n/a**	23	Frequency and procedures for auditing trial conduct, if any, and whether the process will be independent from investigators and the sponsor

For peer review only - http://bmjopen.bmj.com/site/about/guidelines.xhtml

Ethics and dissemination

Research ethics approval **Page 17**	24	Plans for seeking research ethics committee/institutional review board (REC/IRB) approval
Protocol amendments **n/a**	25	Plans for communicating important protocol modifications (eg, changes to eligibility criteria, outcomes, analyses) to relevant parties (eg, investigators, REC/IRBs, trial participants, trial registries, journals, regulators)
Consent or assent **Page 10**	26a	Who will obtain informed consent or assent from potential trial participants or authorised surrogates, and how (see Item 32)
**n/a**	26b	Additional consent provisions for collection and use of participant data and biological specimens in ancillary studies, if applicable
Confidentiality **n/a**	27	How personal information about potential and enrolled participants will be collected, shared, and maintained in order to protect confidentiality before, during, and after the trial
Declaration of interests **Page 18**	28	Financial and other competing interests for principal investigators for the overall trial and each study site
Access to data **Page 18**	29	Statement of who will have access to the final trial dataset, and disclosure of contractual agreements that limit such access for investigators
Ancillary and post-trial care **n/a**	30	Provisions, if any, for ancillary and post-trial care, and for compensation to those who suffer harm from trial participation
Dissemination policy **Page 17**	31a	Plans for investigators and sponsor to communicate trial results to participants, healthcare professionals, the public, and other relevant groups (eg, via publication, reporting in results databases, or other data sharing arrangements), including any publication restrictions
**n/a**	31b	Authorship eligibility guidelines and any intended use of professional writers
**n/a**	31c	Plans, if any, for granting public access to the full protocol, participant-level dataset, and statistical code

Appendices

Informed consent materials	32	Model consent form and other related documentation given to participants and authorised surrogates

Appendix

For peer review only - http://bmjopen.bmj.com/site/about/guidelines.xhtml

Biological specimens	33	Plans for collection, laboratory evaluation, and storage of biological specimens for genetic or molecular analysis in the current trial and for future use in ancillary studies, if applicable
**n/a**		

*It is strongly recommended that this checklist be read in conjunction with the SPIRIT 2013 Explanation & Elaboration for important clarification on the items. Amendments to the protocol should be tracked and dated. The SPIRIT checklist is copyrighted by the SPIRIT Group under the Creative Commons "Attribution-NonCommercial-NoDerivs 3.0 Unported" license.

For peer review only

BMJ Open

Communicating benign biopsy results by telephone in the NHS Breast Screening Programme: a protocol for a cluster randomised crossover trial

Journal:	*BMJ Open*
Manuscript ID	bmjopen-2018-028679.R2
Article Type:	Protocol
Date Submitted by the Author:	11-Jun-2019
Complete List of Authors:	Williamson, Sian; University of Warwick, Health Sciences<br>Johnson, Rebecca; University of Warwick,<br>Sandhu, Harbinder; University of Warwick Warwick Medical School, Clinical Trials Unit<br>Parsons, Nicholas; University of Warwick, Warwick Medical School<br>Jenkins, Jacquie; Public Health England, National Programme Manager – NHS Breast Screening Programme<br>Casey, Margaret; Royal Wolverhampton NHS Trust, Clinical Nurse Specialist Breast Care<br>Kearins, Olive; Public Health England, National Lead Breast Screening QA<br>Taylor-Phillips, Sian; University of Warwick, Warwick Medical School
**Primary Subject Heading**:	Health services research
Secondary Subject Heading:	Communication, Oncology, Public health
Keywords:	PREVENTIVE MEDICINE, Health policy < HEALTH SERVICES ADMINISTRATION & MANAGEMENT, Protocols & guidelines < HEALTH SERVICES ADMINISTRATION & MANAGEMENT, Quality in health care < HEALTH SERVICES ADMINISTRATION & MANAGEMENT

SCHOLARONE™
Manuscripts

Protocol paper V.8 11.06.2019

Communicating benign biopsy results by telephone in the NHS Breast Screening Programme: a protocol for a cluster randomised crossover trial

For peer review only

Sian Williamson, s.williamson@warwick.ac.uk (PhD Student, University of Warwick),

Rebecca Johnson, rebecca.johnson@warwick.ac.uk (Senior Research Fellow, University of Warwick),

Harbinder Sandhu, harbinder.k.sandhu@warwick.ac.uk (Associate Professor, University of Warwick),

Nick Parsons, nick.parsons@warwick.ac.uk (Statistician, Principal Research Fellow, University of Warwick)

Jacquie Jenkins, jacquie.jenkins@phe.gov.uk (National Programme Manager – NHS Breast Screening Programme),

Margaret Casey, margaret.casey@nhs.net (Clinical Nurse Specialist Breast Care),

Olive Kearins, Olive.Kearins@phe.gov.uk (National Lead Breast Screening QA, Public Health England)

& Sian Taylor-Phillips, s.taylor-phillips@warwick.ac.uk (Associate Professor, University of Warwick)

Corresponding author: Sian Taylor-Phillips

Word count (excluding abstract): 3,753 words

Keywords: Mass screening, communication, cancer screening, RCT

For peer review only - http://bmjopen.bmj.com/site/about/guidelines.xhtml

Protocol paper V.8 11.06.2019

Abstract

Introduction – One of the main harms from breast cancer screening is the anxiety caused by false positive results. Various factors may be associated with false-positive anxiety. One modifiable factor may be the method of communication used to deliver results. The aim of this study is to measure the effect on anxiety of receiving benign biopsy results in-person or by telephone.

Methods and analysis – This is a multi-centre cluster randomised crossover trial in the English NHS Breast Screening Programme (NHSBSP) involving repeated survey measures at four time points. Participants will be women of screening age who have a biopsy following a suspicious mammography result, who ultimately receive a benign or normal (B1) result.

Centres will trial both telephone and in-person results on a month-by-month basis, being randomised to which communication method will be trialled first. Women will be blinded to the method of communication they will receive.

The analysis will compare women who have received telephone results and women who have received in-person results. The primary outcome measure will be anxiety (measured by the Psychological Consequences Questionnaire) after receiving results, whilst controlling for baseline anxiety. Secondary outcome measures will include anxiety at 3 and 6 months post-results, understanding of results and patient preferences for how results are communicated. Qualitative telephone interviews will also be conducted to further explore women's reasons for communication preferences. Qualitative and quantitative data will be integrated after initial separate analysis using the Pillar Integration Process.

Ethics and dissemination – This study has been approved by the Public Health England Breast Screening Programme Research Advisory Committee, (BSPRAC_0013, ODR1718_040) and the HRA NHS West Midlands - Coventry & Warwickshire Research Ethics Committee (17/WM/0313). The findings from this study will be disseminated to key stakeholders within the NHSBSP and via academic publications.

Registration details – ISRCTN36997684 https://doi.org/10.1186/ISRCTN36997684

For peer review only - http://bmjopen.bmj.com/site/about/guidelines.xhtml

Protocol paper V.8 11.06.2019

Trial sponsor – This research is part of a PhD award and is funded by the Economic and Social Research Council (ESRC) Doctoral Training Centre at the University of Warwick and Public Health England. The sponsor for this research is Jane Prewett (sponsorship@warwick.ac.uk).

Article summary

Strengths and limitations of this study

- This is the first cluster randomised crossover trial to measure the impact of communication method on patient outcomes when delivering results in breast screening
- The mixed-methods design adds depth to our understanding of communication preferences and the mechanisms by which anxiety may be affected
- The study is in English breast screening centres and generalisability to other contexts should be carefully considered

For peer review only

Protocol paper V.8 11.06.2019

Introduction

The UK National Health Service Breast Screening Programme (NHSBSP) is a population-based screening programme that aims to detect early signs of breast cancer. Asymptomatic women aged 50-70 are invited to attend every three years for a mammogram. The results of the mammogram take a maximum of two weeks as per NHSBSP guidelines (1). If a suspected abnormality is found on the mammogram then the woman is invited back to the clinic for follow-up tests, usually within the next 2 weeks.

Follow-up tests can include clinical examination, a further mammogram or ultrasound. If these indicate suspicion of cancer then the woman receives a core needle biopsy, involving the removal of sections of tissue from the suspicious breast region. This tissue can then be pathologically analysed. Although the procedure is designed to be minimally invasive, leaving only minor bruising, some women find the experience painful and distressing. It is standard practice to perform all follow-up tests (clinical exam, mammogram, ultrasound and biopsy) on the same day in an assessment clinic to avoid unnecessary extra waiting time for patients. Results of diagnostic tests are discussed on a case-by-case basis at multidisciplinary team meetings (MDT) using a triple-assessment of clinical examination, imaging and biopsy report. Clinical guidelines recommend that all follow-up results should be delivered to the woman within one week (2). These results are delivered by either telephone or in-person, depending on the procedure at the breast screening centre.

Screening programmes should provide benefit that outweighs both physical and psychological harm (3, 4). One of the main harms from breast screening is the anxiety caused by false positive results (5). A false-positive result is when a woman has been identified at the screening phase as potentially having cancer, but follow up tests have revealed no abnormalities. Receiving a false-positive result from screening is very common, with the majority of women who are recalled ultimately being given a false-positive result (6). In England, around half of women receive the all clear results at the time of the follow-up tests. Women who are invited to be screened have no symptoms of breast cancer at the time of their initial mammogram. Telling a woman that something suspicious has been found in the mammogram and that further tests are needed can make her feel very anxious and believe that she

For peer review only - http://bmjopen.bmj.com/site/about/guidelines.xhtml

might have cancer (7). For some women, once results confirm the absence of cancer, anxiety declines. However, anxiety can remain elevated for much longer, lasting up to three years after receiving the benign result and leading into the next screening invitation (8). This is an issue, as the NHSBSP has a duty to minimize the harm caused by screening.

There are various factors that may be associated with heightened anxiety during screening such as family history, lower education, younger age and individual differences in personality (1, 9-13). These factors are unmodifiable and tend to be focused at the level of the individual, which makes the minimisation of screening anxiety a challenge. However, it is possible that there are modifiable changes that can be made at the organisational level of screening that may minimise the impact of anxiety. One of these changes is the method of communication used to deliver results.

NHSBSP guidelines for communicating results state that telephone results 'should not be routinely offered' (14). However, most breast screening centres in the UK deliver benign results to the woman over the telephone. Some breast care nurses remain concerned about how the communication method used to deliver results may contribute to the anxiety experienced by women attending screening (2). Telephone results may offer advantages, eliminating the stress and costs associated with transport, parking and anxiously waiting in a clinic for results. However, telephone results eliminates the in-person encounter, meaning all communication is verbal only. Research from other areas has shown that non-verbal communication plays a key role in enhancing understanding and minimising anxiety (15).

The communication methods used to deliver benign results in the NHSBSP has not yet been explored. Therefore, the impact of this communication on women receiving a benign result is unknown.

Aim of the study

The aim of this study is to compare anxiety in women receiving benign biopsy results from the NHSBSP via telephone results or in-person.

For peer review only - http://bmjopen.bmj.com/site/about/guidelines.xhtml

Methods and analysis

Study design

The study design chosen was a multi-centre cluster randomised crossover trial. The randomisation allows for the direct comparison of study outcomes between women who received telephone results and women who received in-person results, whilst controlling for confounding factors such as education, age and individual differences in baseline anxiety.

Patient and public involvement from the charity Independent Cancer Patient Voices was used to guide the design of the study to ensure the acceptability of the method and appropriateness of the participant materials.

Participants and settings

This trial will be conducted in a breast screening centre setting. The study will take place in the UK where women are invited to attend breast screening every 3 years for digital mammography. The study will take place across four time points (see Table 1) with a survey completed by participants at each stage. The study participants will be recruited from 4 English Breast Screening centres across different regions.

Participants will be women between the ages of 47-73 attending the NHSBSP for further tests following a suspicious mammogram. This includes women offered routine screening between ages 50-70 and those receiving extra rounds of screening between the ages of 47-49 or 71-73 as part of the UK age extension trial (16). Women will be recruited at the assessment clinic pre-biopsy. However, only women who have receive a benign (B2) or normal (B1) biopsy result will be included in the longitudinal data collection. Participants will not be included in the study if they presented symptomatically to the breast clinic, if they are not the recommended screening age, if they do not receive a biopsy, if they do not have English as a first or second language and if they do not have the capacity to consent.

For peer review only - http://bmjopen.bmj.com/site/about/guidelines.xhtml

Table 1: Time points for the study

Time point	Sample	Survey content	Method
Time 1 – at assessment clinic	Women attending assessment follow-up clinic	Demographic information<br>Communication preferences<br>Baseline anxiety score (PCQ)<br>Contact details	In-person
Time 2 – after receiving results	Women from time 1 who had a normal or benign biopsy	Communication preferences<br>Repeat anxiety score (PCQ)<br>Measure of understanding	By post
Time 3 – Three month follow-up	Women from time 1 who had a normal or benign biopsy	Repeat anxiety score (PCQ)	By post
Time 4 – Six month follow-up	Women from time 1 who had a normal or benign biopsy	Repeat anxiety score (PCQ)	By post

Measuring anxiety

The Psychological Consequences Questionnaire (PCQ) was selected as the most appropriate measure of anxiety in the breast screening setting (17) and was embedded in the participant surveys (see Appendix 1). The PCQ is a disease specific measure, focusing on breast cancer specific anxiety across 12 questions on three dimensions: emotional, social and physical anxiety. The disease specific measure avoids the contrast-validity that is associated with using generic anxiety measures in the breast screening context (18). The PCQ is widely used in the breast screening setting (1, 8, 19-21).

Participants will rate their anxiety on the PCQ by judging each statement on a scale of 0 (not at all) to 3 (quite a lot of the time). Women will be asked "*Over the last week* how often have you experiences the following things because of *thoughts and feelings about breast cancer*:"

For peer review only

Protocol paper V.8 11.06.2019

Randomisation and blinding

Each centre will be randomised to one of two intervention arms by computer generated random numbers from the trial team base at the University of Warwick (see Table 2). The arm relates to whether the first month at each centre will be telephone or in-person results. Each centre will commence with the communication method randomised in month 1 and continue to alternate between the two communication methods for the duration of the study. This approach allowed for each centre to use both methods of communication, controlling for previous experience (e.g. centres who already telephone are more experienced and therefore women receiving telephone results from this centre might be less anxious).

This approach was selected instead of a block approach, where each centre would deliver 6 months in-person followed by 6 months of telephone. The reason for this was to avoid potential bias from unforeseen centre drop out.

Table 2: Allocation of communication method by arm of the trial

Month	Arm 1	Arm 2
1	Telephone	In-person
2	In-person	Telephone
3	Telephone	In-person
4	In-person	Telephone
5	Telephone	In-person
6	In-person	Telephone

This design was chosen to ensure balance between trial arms at each site whilst addressing practical constraints. Balance between trial arms at each site is important to account for centre level confounders such as staff communication skills and centre level processes.

Individual randomisation was not possible as it interfered with screening centres workflow and fail-safe mechanisms to ensure that every woman was contacted with the correct information within the correct time frame.

Women will be allocated to receive their result based on the date of their attendance at the assessment visit. However, participants will be blinded to the randomisation

For peer review only - http://bmjopen.bmj.com/site/about/guidelines.xhtml

month. Participants will only become aware of the communication method allocated when the results are received (see 'Allocation of communication method' section below for further detail). Breast screening staff will not be blinded, as they will be delivering the results and scheduling the appointments.

Allocation of communication method

There are two types of centres who may be involved in the research: centres who currently deliver benign results in-person and centres who currently deliver benign results by telephone.

For centres currently delivering results in-person, the following process for scheduling a results appointment will be observed: During in-person study months, all consenting women at time 1 will be given an appointment to re-attend to receive their results in-person and will receive the result in-person. During telephone study months, all consenting women at time 1 will be given an appointment to re-attend to receive their results in person. However, they will instead be telephoned prior to their scheduled appointment. Only benign women will be telephoned, with all women receiving other results (e.g. cancer) attending their scheduled appointment

For centres currently delivering results by telephone, the following process for scheduling a results appointment will be observed: All consenting women will be informed that, when their results are ready, they will be contacted by telephone to arrange an appointment to come back for their results. During the 'telephone' months of the study, women will be telephoned as expected. However, instead of arranging an appointment during this telephone call, results will be delivered. This means that, for women who go on to have a cancer result, they can be telephoned to arrange an appointment to attend in-person, as expected. This is in-line with standard practice at these centres. During the in-person months, all consenting women will be informed that, when their results are ready, they will be telephoned to arrange an appointment to come back for their results. These women will be telephoned to arrange an appointment to come back in-person, and at that appointment they will receive their benign/normal result.

For peer review only - http://bmjopen.bmj.com/site/about/guidelines.xhtml

All women not enrolled in the study will receive their screening result based on the current standard practice at the attended centre.

Data collection

Time point 1

Participant recruitment at time one will occur concurrently at each breast screening centre.

Women will be approached during their assessment visit by breast care nurses with Good Clinical Practice (GCP) training. These women will have been recalled from a previous mammogram and may have a biopsy as part of the assessment clinic. The study will be explained to potential participants and nurses will go through the informed consent process. Consenting women will fill out the Time point 1 survey with study responses collected and stored securely before the participant leaves the assessment clinic.

Multi-disciplinary team meeting

At the local level multi-disciplinary team meeting for breast screening staff, women recruited into the study will be included in further time points if they receive a benign (B2) or normal (B1) result. A breast care nurse will compile the contact details of eligible women into a spreadsheet to be sent securely to the research team.

Time point 2

The research team will distribute Time point 2 surveys to eligible women with a pre-paid return envelope. If no response is received within a week, the research team will contact women by telephone as a reminder. A maximum of two telephone contact attempts will be made. This is to ensure anxiety and understanding are measured at the crucial post-results stage of the screening process.

As part of Time point 2 survey, women will be asked if they would like to participate in further research involving an interview about their experience of receiving a screening result.

For peer review only - http://bmjopen.bmj.com/site/about/guidelines.xhtml

Time point 3

The research team will distribute Time point 3 surveys, three months after the biopsy result was received.

Time point 4

The research team will distribute Time point 4 surveys, six months after the biopsy result was received.

Qualitative telephone interviews

The qualitative telephone interviews will explore why women prefer certain methods of communication. Women will be recruited from the time point 2 survey. Women who express an interest in participating will be sent further information about the interviews. If they wish to participate, women will return the consent form in the pre-paid envelope. Women will then be contacted by telephone by the research team to be interviewed.

The semi-structured telephone interviews will each take 10-20 minutes. Questions will encompass the woman's experience of receiving a result from screening. This will involve asking how the woman felt, whether she understood her result and an exploration of her views on different methods for communicating results. Interviews will be audio-recorded and transcribed verbatim. Data collection will cease once no further themes emerge and data saturation is reached.

Mixed methods integration

Using a mixed methods approach, the quantitative preference survey data will be combined with the findings from the qualitative interviews. The quantitative data asks a binary choice question regarding women's communication preferences (telephone or in-person), whilst the qualitative interviews expand on this by exploring how women justify certain preferences for communication. In mixing the data, the qualitative data will be used to *expand* the understanding of the findings from the quantitative surveys (22). Expansion provides richness and detail, expanding why and how women form communication preferences and moving understanding of preferences *beyond* what quantitative data or qualitative data in isolation can

For peer review only - http://bmjopen.bmj.com/site/about/guidelines.xhtml

elucidate. The value added by integrating the knowledge of both *what* women prefer and *why* is in the completeness of our understanding of preferences, making the evidence that will inform the NHSBSP policy decisions comprehensive and patient-centred.

For peer review only

Sample size considerations

In order to determine the sample size for a clustered randomised cross-over study, a full specification of the important within-cluster (centre) between-period and within-period correlations is required (23). There is currently no available evidence on the magnitude of these correlations in our selected setting, so rather than arbitrarily selecting values we adopt a conservative approach and assume that the (within-centre) between-period correlations are zero and proceed to power as if the design were a cluster randomised design. Although the cross-over aspect of the design is not explicitly accounted for in the sample size calculation, it is incorporated fully in the analysis of the primary study outcome (PCQ anxiety score at time-point 2). The aim of the study to be able to detect a clinically significant difference of 3 points in the PCQ (the difference between the score on one statement being 0, not at all, and 3, quite a lot of the time).

[Insert Figure 1 here]

Figure 1: Flow of required sample size for the study

Assuming the primary outcome is approximately normally distributed, and the test is at the 5% significance level with 80% power to detect an effect of the specified size, 194 participants are required at time 2 i.e. 97 participants per arm. Allowing for attrition rate between time point 1 and time point 2 due to participant withdrawal (15%) and participant eligibility (50%), a total of 457 participants will be recruited at time point 1. Participant withdrawal was calculated based upon a mean response rate of 60% from previous research using postal surveys in a medical setting (24) with a loss of 15% at each time point as a conservative estimate.

In order to account for clustering due to the recruiting centre, design effect was applied that inflated the sample size. The intra-cluster (within-centre) correlation coefficient (ICC) was set to be 0.01 and the number of observations within each cluster were assumed to be equal. With the sample size of 194 women, divided by the number of centres (4) and then divided by the number of interventions (2), this led to the number of observations within a cluster being 48.2. This gave a design

For peer review only - http://bmjopen.bmj.com/site/about/guidelines.xhtml

effect of 1.49. Taking this into account, the sample size needed to achieve statistical significance (194 women) was multiplied by the design effect, giving a total sample of 290 women at time point 2 when rounded up (see Figure 1).

31,926 women are recalled for a biopsy each year. If half of the 31,926 biopsies come back as benign, this leaves a potential sample of 15,963. This means that, on average across 80 breast screening centres, each centre will have around 200 benign biopsy results each year. Therefore, assuming 50% participation rates of eligible women, recruitment will require four centres for one year.

For peer review only

Outcomes and study measures (primary outcome, secondary outcomes)

Primary outcome

The primary outcome is the PCQ anxiety score at time point 2.

A comparison in anxiety score will be made between women who receive results in-person and women who receive results over the telephone.

Secondary outcomes

Secondary outcomes are:

- PCQ anxiety score at 3 month follow-up (time 3)
- PCQ anxiety score at 6 month follow-up (time 4)
- Subjective understanding of results (time 2)
  - Measured using a survey question designed in collaboration with NHSBSP stakeholders
- Objective understanding of results (time 2)
  - Measured using a survey question designed in collaboration with NHSBSP stakeholders
- Quantitative preferences for results communication before results (time 1)
- Quantitative preferences for results communication after results (time 2)
- Qualitative preferences for results communication before results (time 1)
- Qualitative preferences for results communication after results (time 2)

For peer review only - http://bmjopen.bmj.com/site/about/guidelines.xhtml

Protocol paper V.8 11.06.2019

Analysis

Quantitative data - Statistical analysis

A formal and more detailed statistical analysis plan (SAP) will be developed by the trial team prior to the completion of recruitment.

Primary outcome

The primary analysis will use a mixed effects linear regression model to estimate the effects of communication method on anxiety (time 2), after adjusting for baseline anxiety (time 1). PCQ score will be treated as a continuous variable and to be approximately normally distributed for all analyses.

The model set-up and fixed and random effects are as follows:

- Response variable – Anxiety at time 2 (PCQ)
- Baseline – Anxiety at time 1 (PCQ)
- Fixed explanatory effects (model covariates) – age, ethnicity, previous attendance, previous biopsy, education, marital status.
- Random effects – centre and temporal (period) effects
- Comparator variable - Method of communication received – telephone or in-person

Statistical significance will be assessed at the 5% level.

Secondary outcomes

Longitudinal anxiety scores at 3 and 6 months will be analysed in the same way as the primary anxiety outcome.

Differences in understanding score between communication methods groups will be assessed using a logistic regression model, adjusting for fixed effects.

- Outcome variable – Subjective understanding score (binary – yes or no), objective understanding score (binary – right or wrong)
- Fixed effects – age, ethnicity, previous attendance, previous biopsy, education, marital status.
- Comparator variable - Method of communication received (telephone/in-person)

For peer review only - http://bmjopen.bmj.com/site/about/guidelines.xhtml

Protocol paper V.8 11.06.2019

Preference data from the quantitative surveys will be presented in the form of percentages. All analyses will be implemented using IBM SPSS Statistics 25.

Qualitative data analysis

Qualitative preference data from the time point 1 and time point 2 surveys and data from the telephone interviews will be analysed using inductive thematic analysis (25), managed using NVivo10.

Mixed-methods integration

To integrate the quantitative and qualitative preference data, the Pillar Integration Process will be used (26). This analytical integration technique uses four systematic stages (listing, matching, checking and pillar-building) to identify and examine connections and discrepancies in qualitative and quantitative findings. It allows for the visual display of the data and findings; this enhances overall transparency of the integration approach and the results of such an integration.

For peer review only - http://bmjopen.bmj.com/site/about/guidelines.xhtml

Ethics

Attention was given to the various ethical challenges of the trial. The main ethical issue will be the use of sensitive patient information (addresses, telephone numbers). Participants will be told explicitly how their contact details will be used and stored throughout the data collection process. Participants will give informed consent for their contact details to be used for the purposes of the study.

All electronic data will be transferred securely in a password protected excel document from secure email accounts. All raw survey data will be collected directly from the centres by the lead researcher and be transferred in a secure lock-box.

Ethical approval for this study was granted by the following organisations:

- Public Health England Breast Screening Programme Research Advisory Committee, BSPRAC_0013 (ODR1718_040)
- HRA NHS West Midlands - Coventry & Warwickshire Research Ethics Committee (17/WM/0313)

Dissemination

Results from the trial will disseminated directly to key stakeholders within the NHSBSP. This will encourage discussion regarding how benign results are communicated in breast screening, and how this might best be implemented in order to minimise the anxiety women experience.

The results will also be disseminated via academic publications.

For peer review only - http://bmjopen.bmj.com/site/about/guidelines.xhtml

Protocol paper V.8 11.06.2019

Current study status

The trial began recruitment in February 2018. Data collection is due to conclude in March 2019. The trial statisticians (SW and NP) have received recruitment data but no results will be transferred to the statisticians until recruitment is closed in March 2019.

Author contributions

SZW is the lead researcher. SZW and STP drafted the manuscript. SZW, RJ, HS and STP participated in the design of the study. JJ, MC and OK are key stakeholders in the NHSBSP who assisted with the study design. NP aided with the statistical analysis. All authors have reviewed and approved the manuscript.

Funding statement

This research is part of a PhD award and is funded by the Economic and Social Research Council (ESRC) Doctoral Training Centre at the University of Warwick. The funding has been awarded for this studentship to Sian Williamson for her PhD project for 4 years of full-time study. The award consists of payment of academic fees and a maintenance award.

A further contract between the University of Warwick, Public Health England (PHE) and the PhD Student (Sian Williamson) has secured £4,000 in research expenses.

Competing interests

None declared

For peer review only

Protocol paper V.8 11.06.2019

References

1. Brett J, Bankhead C, Henderson B, Watson E, Austoker J. The psychological impact of mammographic screening. A systematic review. Psycho-Oncology. 2005;14(11):917-38.
2. Chapman K. Interim quality assurance guidelines for clinical nurse specialists in breast cancer screening. 2012.
3. Raffle AE, Gray JM. Screening: evidence and practice: Oxford University Press; 2007.
4. Marmot MG, Altman D, Cameron D, Dewar J, Thompson S, Wilcox MJBjoc. The benefits and harms of breast cancer screening: an independent review. 2013;108(11):2205.
5. Bond M, Pavey T, Welch K, Cooper C, Garside R, Dean S, et al. Systematic review of the psychological consequences of false-positive screening mammograms. 2013.
6. DeFrank JT, Brewer N. A model of the influence of false-positive mammography screening results on subsequent screening. Health psychology review. 2010;4(2):112-27.
7. Solbjør M, Forsmo S, Skolbekken J-A, Sætnan AR. Experiences of recall after mammography screening—a qualitative study. Health care for women international. 2011;32(11):1009-27.
8. Brett J, Austoker J. Women who are recalled for further investigation for breast screening: psychological consequences 3 years after recall and factors affecting re-attendance. Journal of Public Health. 2001;23(4):292-300.
9. Matteau TM. Psychology and screening: narrowing the gap between efficacy and effectiveness. British Journal of Clinical Psychology. 1994;33(1):1-10.
10. Ando N, Iwamitsu Y, Kuranami M, Okazaki S, Nakatani Y, Yamamoto K, et al. Predictors of psychological distress after diagnosis in breast cancer patients and patients with benign breast problems. Psychosomatics. 2011;52(1):56-64.
11. Keyzer-Dekker CM, de Vries J, Mertens MC, Roukema JA, van der Steeg AF. Cancer or no cancer: the influence of trait anxiety and diagnosis on quality of life with breast cancer and benign disease: a prospective, longitudinal study. World journal of surgery. 2013;37(9):2140-7.
12. Montgomery M, McCrone SH. Psychological distress associated with the diagnostic phase for suspected breast cancer: systematic review. Journal of advanced nursing. 2010;66(11):2372-90.
13. Sweeny K, Andrews SE. Mapping individual differences in the experience of a waiting period. Journal of personality and social psychology. 2014;106(6):1015.
14. England. PH. NHS public health functions agreement 2017- 18 Service specification no.24 Breast Screening Programme. In: England. PH, editor. 2017.
15. Knapp ML, Hall J. Nonverbal behavior in human interaction. Wadsworth: New York; 2002.
16. 2000. CgIBMNLoMUF. Identifier NCT01081288; Extending the National Health Service (NHS) Breast Screening Age Range (AgeX). 5 March 2010 (16 November 2018).
17. Cockburn J, De Luise T, Hurley S, Clover K. Development and validation of the PCQ: a questionnaire to measure the psychological consequences of screening mammography. Social science & medicine. 1992;34(10):1129-34.

For peer review only - http://bmjopen.bmj.com/site/about/guidelines.xhtml

18. Brodersen J, Thorsen H, Cockburn J. The adequacy of measurement of short and long-term consequences of false-positive screening mammography. Journal of medical screening. 2004;11(1):39-44.
19. Sweeny K, Christianson D, McNeill J. The Psychological Experience of Awaiting Breast Diagnosis. 2018.
20. Cockburn J, Staples M, Hurley SF, De Luise T. Psychological consequences of screening mammography. Journal of Medical Screening. 1994;1(1):7-12.
21. Brewer NT, Salz T, Lillie SE. Systematic Review: The Long-Term Effects of False-Positive MammogramsThe Long-Term Effects of False-Positive Mammograms. Annals of internal medicine. 2007;146(7):502-10.
22. Creswell JW, Klassen AC, Plano Clark VL, Smith KC. Best practices for mixed methods research in the health sciences. Bethesda (Maryland): National Institutes of Health. 2011:2094-103.
23. Arnup SJ, McKenzie JE, Hemming K, Pilcher D, Forbes ABJT. Understanding the cluster randomised crossover design: a graphical illustration of the components of variation and a sample size tutorial. 2017;18(1):381.
24. Asch DA, Jedrziewski MK, Christakis NAJJoce. Response rates to mail surveys published in medical journals. 1997;50(10):1129-36.
25. Braun V, Clarke VJQrip. Using thematic analysis in psychology. 2006;3(2):77-101.
26. Johnson RE, Grove AL, Clarke AJJoMMR. Pillar Integration Process: A Joint Display Technique to Integrate Data in Mixed Methods Research. 2017:1558689817743108.

For peer review only

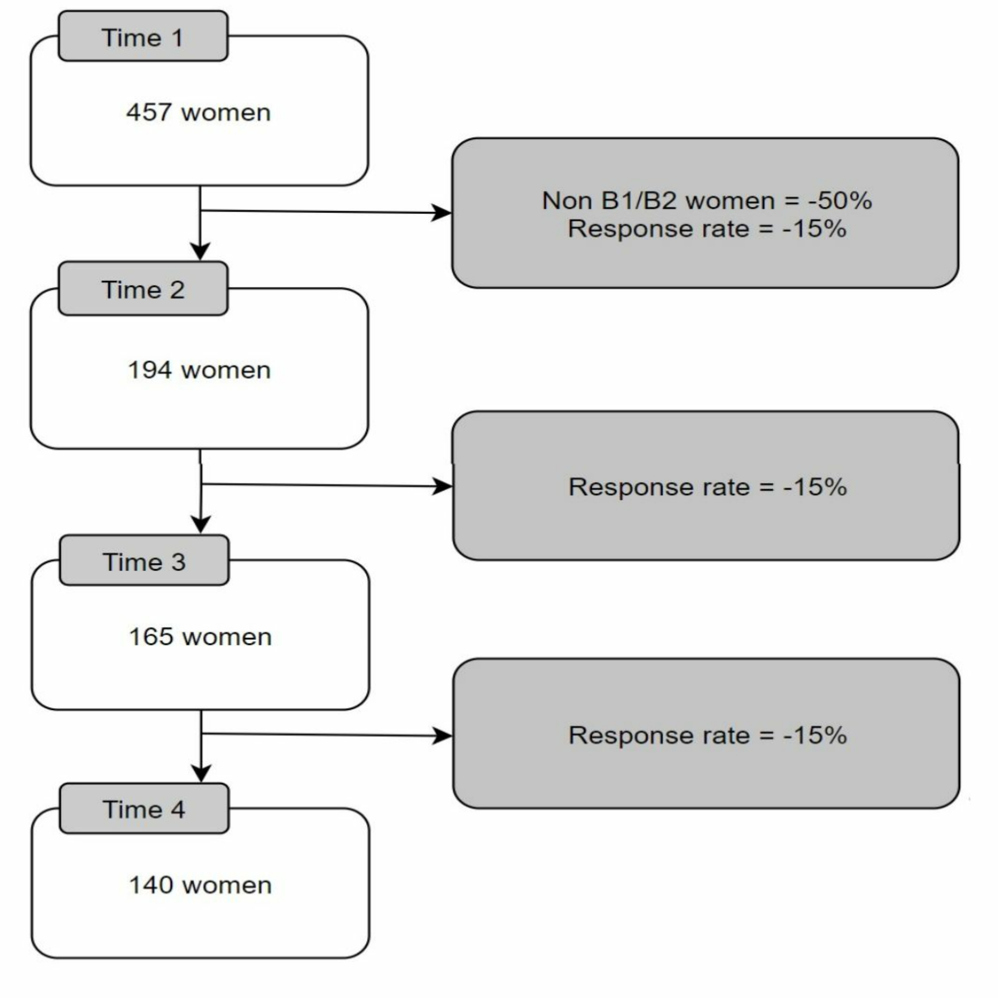

Figure 1 - Flow of required sample size for the study

89x89mm (300 x 300 DPI)

Survey (time 1) – V.6 05.04.2018

Communicating results in breast screening: Research survey – Time 1

Public Health England

WARWICK THE UNIVERSITY OF WARWICK

NHS Cancer Screening Programmes

PARTICIPANT NAME: ______________________________

(Please ensure this is completed)

For researcher use only:

Participant ID number: ______________________________

B1 or B2 result:

- ☐ Yes
- ☐ No

For peer review only - http://bmjopen.bmj.com/site/about/guidelines.xhtml

Survey (time 1) – V.6 05.04.2018

Participant information

1) What is your age?

___________ years

2) Have you attended screening before?

☐ Yes

☐ No

2a) If yes, did you have a biopsy (a needle inserted into the breast to remove tissue)?

☐ Yes

☐ No

☐ Can't remember

3) Please tick the highest level of education you have achieved:

☐ No schooling completed

☐ High school or equivalent

☐ Some college

☐ Bachelor's degree

☐ Master's degree

☐ Doctoral degree

☐ Professional degree

☐ Other

4) Please tick your marital status:

☐ Single

☐ Domestic partnership

☐ Married

☐ Divorced

☐ Widowed

☐ Separated

5) What is your ethnic group?

White:

☐ English/Welsh/Scottish/ Northern Irish/British

☐ Irish

☐ Gypsy or Irish Traveller

☐ Any other White background

Asian/Asian British:

☐ Indian

☐ Pakistani

☐ Bangladeshi

☐ Chinese

☐ Any other Asian background

Mixed/multiple ethnic groups:

☐ White and Black Caribbean

☐ White and Black African

☐ White and Asian

☐ Any other Mixed/Multiple ethnic background

Black/African/Caribbean/Black British:

☐ African

☐ Caribbean

☐ Any other Black/African/Caribbean background

Other ethnic group:

☐ Arab

☐ Any other ethnic group

For peer review only

Preferences for communication

When a screening result is not cancer, some centres deliver these results in-person and some deliver these results over the telephone. We want to know how you would prefer to receive screening results, *if you were given the choice.*

Please note, if you tick a preference, you may not necessarily get your result delivered this way.

6) How would you prefer to receive your results from screening?

☐ In-person at the screening centre
☐ By telephone

7) Why would you prefer to receive your results this way?

Please write a brief explanation:

____
____
____

For peer review only

For peer review only - http://bmjopen.bmj.com/site/about/guidelines.xhtml

Survey (time 1) – V.6 05.04.2018

How do you feel?

8) *Over the last week* how often have you experiences the following things because of *thoughts and feelings about breast cancer*:

	Not at all	Rarely	Some of the time	Quite a lot of the time
Had trouble sleeping	0	1	2	3
Experienced a change in appetite	0	1	2	3
Been unhappy or depressed	0	1	2	3
Been scared and panicky	0	1	2	3
Felt nervous or strung up	0	1	2	3
Felt under strain	0	1	2	3
Found you have been keeping things from those who are close to you	0	1	2	3
Found yourself taking things out on other people	0	1	2	3
Found yourself noticeably withdrawing from those who are close to you	0	1	2	3
Had difficultly doing things around the house that you normally do	0	1	2	3
Had difficulty meeting work or other commitments	0	1	2	3
Felt worried about your future	0	1	2	3

For peer review only

9) Please provide a contact name and address for follow-up surveys to be sent:

(Please note that this address will remain anonymous and all contact information will be destroyed once the study is complete. This address will only be used for the purposes of this research to post follow-up surveys.)

____

____

____

____

____

10) Please provide a contact telephone number:

(Please note that this contact number will remain anonymous and all contact information will be destroyed once the study is complete. This telephone number will only be used for the purposes of this research to remind you of the first follow-up survey.)

____

11) Would you like to hear about the results from this research?

(If you choose yes, you will be sent a copy of the results from this research via the address provided)

- ☐ Yes
- ☐ No

For peer review only

SPIRIT

STANDARD PROTOCOL ITEMS: RECOMMENDATIONS FOR INTERVENTIONAL TRIALS

SPIRIT 2013 Checklist: Recommended items to address in a clinical trial protocol and related documents*

Section/item	Item No	Description
Title<br>**Page 1**	1	Descriptive title identifying the study design, population, interventions, and, if applicable, trial acronym
Trial registration<br>**Page 2**	2a	Trial identifier and registry name. If not yet registered, name of intended registry
**n/a**	2b	All items from the World Health Organization Trial Registration Data Set
Protocol version<br>***Top of page***	3	Date and version identifier
Funding<br>**Page 18**	4	Sources and types of financial, material, and other support
Roles and responsibilities<br>**Page 18**	5a	Names, affiliations, and roles of protocol contributors
**Page 3**	5b	Name and contact information for the trial sponsor
**n/a - No role in these activities**	5c	Role of study sponsor and funders, if any, in study design; collection, management, analysis, and interpretation of data; writing of the report; and the decision to submit the report for publication, including whether they will have ultimate authority over any of these activities
**n/a**	5d	Composition, roles, and responsibilities of the coordinating centre, steering committee, endpoint adjudication committee, data management team, and other individuals or groups overseeing the trial, if applicable (see Item 21a for data monitoring committee)

For peer review only

Introduction

Background and rationale **Page 4**	6a	Description of research question and justification for undertaking the trial, including summary of relevant studies (published and unpublished) examining benefits and harms for each intervention
**n/a**	6b	Explanation for choice of comparators
Objectives **Page 6**	7	Specific objectives or hypotheses
Trial design **Page 6**	8	Description of trial design including type of trial (eg, parallel group, crossover, factorial, single group), allocation ratio, and framework (eg, superiority, equivalence, noninferiority, exploratory)

Methods: Participants, interventions, and outcomes

Study setting **Page 6**	9	Description of study settings (eg, community clinic, academic hospital) and list of countries where data will be collected. Reference to where list of study sites can be obtained
Eligibility criteria **Page 6**	10	Inclusion and exclusion criteria for participants. If applicable, eligibility criteria for study centres and individuals who will perform the interventions (eg, surgeons, psychotherapists)
Interventions **Page 8**	11a	Interventions for each group with sufficient detail to allow replication, including how and when they will be administered
**n/a**	11b	Criteria for discontinuing or modifying allocated interventions for a given trial participant (eg, drug dose change in response to harms, participant request, or improving/worsening disease)
**n/a**	11c	Strategies to improve adherence to intervention protocols, and any procedures for monitoring adherence (eg, drug tablet return, laboratory tests)
**n/a**	11d	Relevant concomitant care and interventions that are permitted or prohibited during the trial

For peer review only - http://bmjopen.bmj.com/site/about/guidelines.xhtml

Outcomes **Page 14**	12	Primary, secondary, and other outcomes, including the specific measurement variable (eg, systolic blood pressure), analysis metric (eg, change from baseline, final value, time to event), method of aggregation (eg, median, proportion), and time point for each outcome. Explanation of the clinical relevance of chosen efficacy and harm outcomes is strongly recommended
Participant timeline **Page 10**	13	Time schedule of enrolment, interventions (including any run-ins and washouts), assessments, and visits for participants. A schematic diagram is highly recommended (see Figure)
Sample size **Page 13**	14	Estimated number of participants needed to achieve study objectives and how it was determined, including clinical and statistical assumptions supporting any sample size calculations
Recruitment **Page 13**	15	Strategies for achieving adequate participant enrolment to reach target sample size

Methods: Assignment of interventions (for controlled trials)

Allocation:

Sequence generation **Page 8**	16a	Method of generating the allocation sequence (eg, computer-generated random numbers), and list of any factors for stratification. To reduce predictability of a random sequence, details of any planned restriction (eg, blocking) should be provided in a separate document that is unavailable to those who enrol participants or assign interventions
Allocation concealment mechanism **n/a**	16b	Mechanism of implementing the allocation sequence (eg, central telephone; sequentially numbered, opaque, sealed envelopes), describing any steps to conceal the sequence until interventions are assigned
Implementation **Page 8**	16c	Who will generate the allocation sequence, who will enrol participants, and who will assign participants to interventions
Blinding (masking) **Page 8**	17a	Who will be blinded after assignment to interventions (eg, trial participants, care providers, outcome assessors, data analysts), and how
**n/a**	17b	If blinded, circumstances under which unblinding is permissible, and procedure for revealing a participant's allocated intervention during the trial

For peer review only - http://bmjopen.bmj.com/site/about/guidelines.xhtml

Methods: Data collection, management, and analysis

Data collection methods **Page 15**	18a	Plans for assessment and collection of outcome, baseline, and other trial data, including any related processes to promote data quality (eg, duplicate measurements, training of assessors) and a description of study instruments (eg, questionnaires, laboratory tests) along with their reliability and validity, if known. Reference to where data collection forms can be found, if not in the protocol
**n/a**	18b	Plans to promote participant retention and complete follow-up, including list of any outcome data to be collected for participants who discontinue or deviate from intervention protocols
Data management **n/a**	19	Plans for data entry, coding, security, and storage, including any related processes to promote data quality (eg, double data entry; range checks for data values). Reference to where details of data management procedures can be found, if not in the protocol
Statistical methods **Page 15**	20a	Statistical methods for analysing primary and secondary outcomes. Reference to where other details of the statistical analysis plan can be found, if not in the protocol
**Page 15**	20b	Methods for any additional analyses (eg, subgroup and adjusted analyses)
**n/a**	20c	Definition of analysis population relating to protocol non-adherence (eg, as randomised analysis), and any statistical methods to handle missing data (eg, multiple imputation)

Methods: Monitoring

Data monitoring **n/a**	21a	Composition of data monitoring committee (DMC); summary of its role and reporting structure; statement of whether it is independent from the sponsor and competing interests; and reference to where further details about its charter can be found, if not in the protocol. Alternatively, an explanation of why a DMC is not needed
**n/a**	21b	Description of any interim analyses and stopping guidelines, including who will have access to these interim results and make the final decision to terminate the trial
Harms **n/a**	22	Plans for collecting, assessing, reporting, and managing solicited and spontaneously reported adverse events and other unintended effects of trial interventions or trial conduct
Auditing **n/a**	23	Frequency and procedures for auditing trial conduct, if any, and whether the process will be independent from investigators and the sponsor

Ethics and dissemination

Research ethics approval **Page 17**	24	Plans for seeking research ethics committee/institutional review board (REC/IRB) approval
Protocol amendments **n/a**	25	Plans for communicating important protocol modifications (eg, changes to eligibility criteria, outcomes, analyses) to relevant parties (eg, investigators, REC/IRBs, trial participants, trial registries, journals, regulators)
Consent or assent **Page 10**	26a	Who will obtain informed consent or assent from potential trial participants or authorised surrogates, and how (see Item 32)
**n/a**	26b	Additional consent provisions for collection and use of participant data and biological specimens in ancillary studies, if applicable
Confidentiality **n/a**	27	How personal information about potential and enrolled participants will be collected, shared, and maintained in order to protect confidentiality before, during, and after the trial
Declaration of interests **Page 18**	28	Financial and other competing interests for principal investigators for the overall trial and each study site
Access to data **Page 18**	29	Statement of who will have access to the final trial dataset, and disclosure of contractual agreements that limit such access for investigators
Ancillary and post-trial care **n/a**	30	Provisions, if any, for ancillary and post-trial care, and for compensation to those who suffer harm from trial participation
Dissemination policy **Page 17**	31a	Plans for investigators and sponsor to communicate trial results to participants, healthcare professionals, the public, and other relevant groups (eg, via publication, reporting in results databases, or other data sharing arrangements), including any publication restrictions
**n/a**	31b	Authorship eligibility guidelines and any intended use of professional writers
**n/a**	31c	Plans, if any, for granting public access to the full protocol, participant-level dataset, and statistical code

Appendices

Informed consent materials	32	Model consent form and other related documentation given to participants and authorised surrogates

Appendix

For peer review only - http://bmjopen.bmj.com/site/about/guidelines.xhtml

Biological specimens	33	Plans for collection, laboratory evaluation, and storage of biological specimens for genetic or molecular analysis in the current trial and for future use in ancillary studies, if applicable

n/a

*It is strongly recommended that this checklist be read in conjunction with the SPIRIT 2013 Explanation & Elaboration for important clarification on the items. Amendments to the protocol should be tracked and dated. The SPIRIT checklist is copyrighted by the SPIRIT Group under the Creative Commons "Attribution-NonCommercial-NoDerivs 3.0 Unported" license.

For peer review only
